# Mitochondrial-Shaping Proteins in Cardiac Health and Disease – the Long and the Short of It!

**DOI:** 10.1007/s10557-016-6710-1

**Published:** 2017-02-11

**Authors:** Sang-Bing Ong, Siavash Beikoghli Kalkhoran, Sauri Hernández-Reséndiz, Parisa Samangouei, Sang-Ging Ong, Derek John Hausenloy

**Affiliations:** 1 Cardiovascular and Metabolic Disorders Program, Duke-National University of Singapore Medical School, 8 College Road, Singapore, 169857 Singapore; 2 National Heart Research Institute Singapore, National Heart Centre Singapore, Singapore, Singapore; 3 The Hatter Cardiovascular Institute, Institute of Cardiovascular Science, University College London, London, UK; 4 Stanford Cardiovascular Institute, Stanford University School of Medicine, Stanford, CA USA; 5 The National Institute of Health Research, University College London Hospitals Biomedical Research Centre, London, UK

**Keywords:** Mitochondrial morphology, Ischemia/reperfusion injury, Mitochondrial fusion, Mitochondrial fission, Mfn1, OPA1, Mfn2, Drp1

## Abstract

Mitochondrial health is critically dependent on the ability of mitochondria to undergo changes in mitochondrial morphology, a process which is regulated by mitochondrial shaping proteins. Mitochondria undergo fission to generate fragmented discrete organelles, a process which is mediated by the mitochondrial fission proteins (Drp1, hFIS1, Mff and MiD49/51), and is required for cell division, and to remove damaged mitochondria by mitophagy. Mitochondria undergo fusion to form elongated interconnected networks, a process which is orchestrated by the mitochondrial fusion proteins (Mfn1, Mfn2 and OPA1), and which enables the replenishment of damaged mitochondrial DNA. In the adult heart, mitochondria are relatively static, are constrained in their movement, and are characteristically arranged into 3 distinct subpopulations based on their locality and function (subsarcolemmal, myofibrillar, and perinuclear). Although the mitochondria are arranged differently, emerging data supports a role for the mitochondrial shaping proteins in cardiac health and disease. Interestingly, in the adult heart, it appears that the pleiotropic effects of the mitochondrial fusion proteins, Mfn2 (endoplasmic reticulum-tethering, mitophagy) and OPA1 (cristae remodeling, regulation of apoptosis, and energy production) may play more important roles than their pro-fusion effects. In this review article, we provide an overview of the mitochondrial fusion and fission proteins in the adult heart, and highlight their roles as novel therapeutic targets for treating cardiac disease.

## Introduction to Mitochondrial Morphology

Mitochondrial health is critically dependent on the ability of mitochondria to move and change their morphology. By undergoing fission they generate fragmented discrete mitochondria, a process which is regulated by the mitochondrial fission proteins, dynamic-related peptide-1 (Drp1), human fission protein-1 (hFis1), mitochondrial fission factor (Mff) and mitochondrial dynamics proteins 49 and 51 (MiD49 and 51). Mitochondrial fission is essential for cell division and is required to remove damaged mitochondria by mitophagy. In contrast, the fusion of mitochondria generates elongated interconnected networks, a process which is orchestrated by the mitochondrial fusion proteins, Mitofusins 1 and 2 (Mfn1 and Mfn2), and optic atrophy protein-1 (OPA1), thereby enabling the replenishment of damaged mitochondrial DNA [1] or facilitation of intracellular energy distribution [2].

**Fig. 1:**
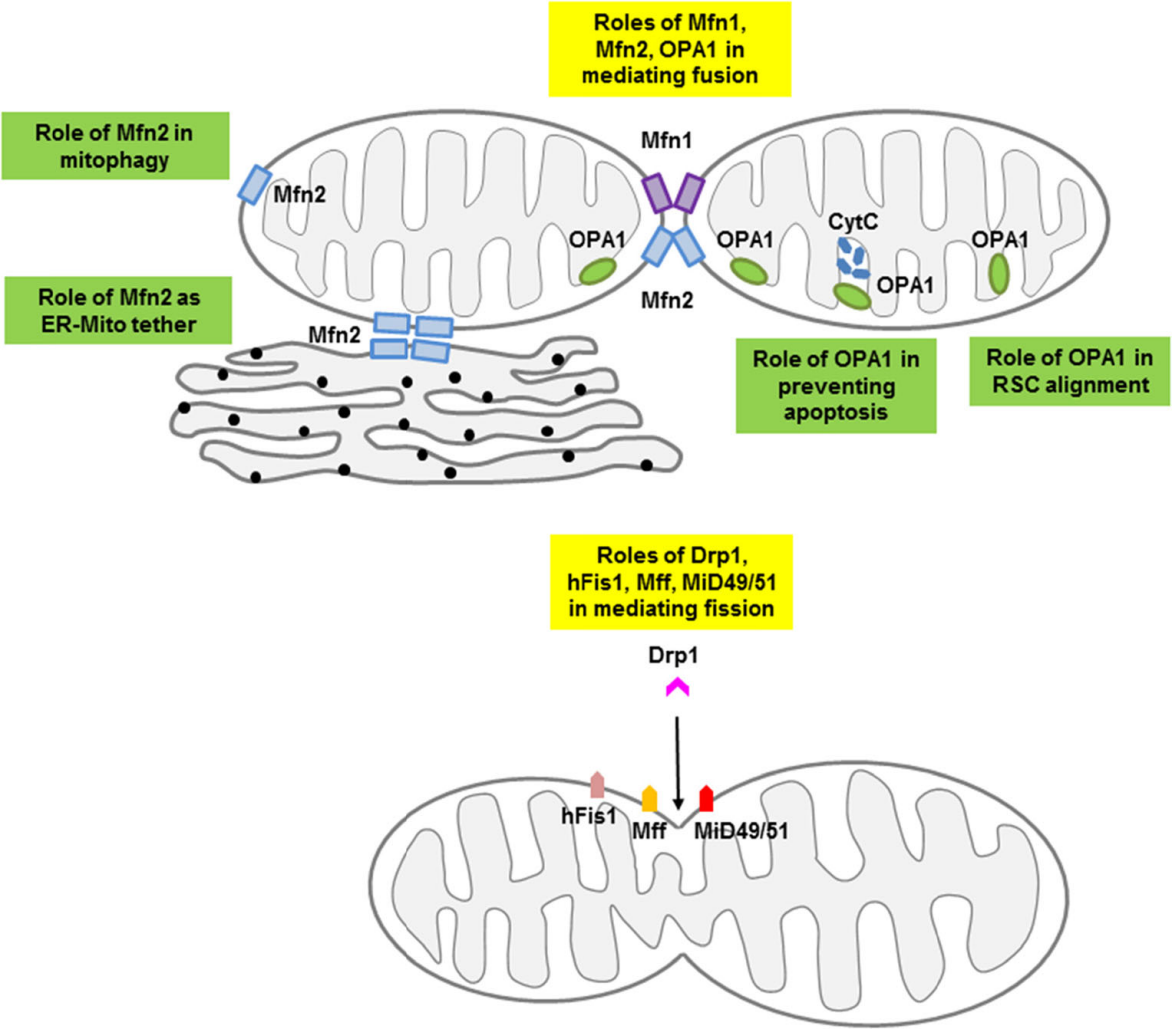
Diagram depicting interactions of the mitochondrial-shaping proteins. The pro-fusion proteins – Mfn1, Mfn2 and OPA1 function to fuse adjacent mitochondria while Drp1 interacts with the docking proteins – Mff, Fis1 or MiD49/51 to induce mitochondrial fission. In addition to their profusion effect, Mfn2 and OPA1 have pleiotropic non-fusion actions. RSC, respiratory supercomplex; ER, endoplasmic reticulum; Mito, mitochondria

Until recently, the investigation of mitochondrial morphology had been largely confined to non-cardiac cells, in which mitochondria have unrestricted movements and are distributed throughout the cytoplasm into tubular networks. In contrast, in the adult heart, mitochondria are relatively immobile, are constrained in their ability to move, and are distributed into 3 distinct subpopulations based on their locality and function (subsarcolemmal, myofibrillar, and perinuclear). Although the mitochondria are arranged differently, emerging data supports a role for the mitochondrial shaping proteins in cardiac health and disease, with their effects on mediating changes in mitochondrial morphology playing a dominant role [3–6]. Interestingly, although the roles of the mitochondrial fission proteins are closely related to their effects on mitochondrial morphology in the adult heart, the mitochondrial fusion proteins appear to have a number of pleiotropic non-fusion roles, which may play more important roles in cardiac health and disease than their pro-fusion effects (see Fig. 1). Mfn2 has been reported to act as a tethering protein between mitochondria and endoplasmic reticulum (ER) [7, 8], and to be a critical mediator of mitophagy [9, 10]. Through its effects on cristae remodeling, OPA1 has been shown to regulate mitochondrial cytochrome C release and propensity to apoptosis, and facilitate mitochondrial energy production via its effects on the respiratory supercomplexes. In this review article, we provide an overview of the mitochondrial shaping proteins in the adult heart, and highlight their roles as novel therapeutic targets for treating cardiac disease. For more detailed general reviews on mitochondrial morphology please refer to the following articles [11–16].

## Mitochondrial Fission Proteins

The process of mitochondrial fission requires the activation and dephosphorylation of cytosolic Drp1, which allows the latter to translocate to mitochondria, and bind to docking receptors on the outer mitochondrial membrane (OMM). Here, Drp1 oligomerises and forms a spiral around the mitochondrion, constricting it into two halves [17–20]. The roles the other components of the mitochondrial fission machinery, hFis1, Mff and MiD49/51, play in this process are currently under investigation. The current evidence suggests that Mff [21–25] and MiD49/51 [22, 26–31] act as docking proteins on the OMM for Drp1, and that hFis1 plays a regulatory role in mediating mitochondrial fission [32, 33]. Interestingly, Mff and MiD49/51 have been reported to exert contrasting effects on Drp1 GTPase activity at the OMM, with the former stimulating, and the latter inhibiting Drp1 GTPase activity [22] - the resultant effect being dependant on the stoichiometric levels of the adaptors as well as the presence of other signals [22]. Another layer of complexity is added with the existence of different subpopulations of Drp1 in the cytosol (dimers or tetramers or high order equilibrium) [34–36] and even multiple splice variants of Drp1 [37], whereby recruitment of specific subpopulations of Drp1 by either Mff or MiD49/51 result in different effects on mitochondrial morphology [23, 38]. A number of other proteins have been shown to regulate mitochondrial fission in non-cardiac cells including COX assembly factor ccdc56 [39], the OMM protein FUNDC1 [40–42] and the myeloid cell leukemia factor 1 (Mcl-1) protein [43].

The ER and cytoskeleton have recently been demonstrated to contribute to the mitochondrial fission process. Actin polymerisation by the ER-associated inverted formin-2 (INF2) [44–46] has been shown to provide the force required for partial constriction of the mitochondria, an event which has been reported to facilitate the translocation of cytosolic Drp1 to these pre-constriction contact sites in the OMM. Drp1 translocation from the cytosol to the OMM has also been demonstrated to be mediated by other components of the cytoskeleton including septins [47]. Transient de novo assembly of F-actin on the OMM has been shown to mediate Drp1-mediated fission [48], in a manner that is synergistic with Mff [49]. Inhibiting actin polymerization, myosin IIA, or the formin INF2 can also reduce this Drp1/actin interaction [49].

The regulation of Drp1 activity is under the control of a large number of post-translational modifications including SUMOylation [50], phosphorylation [51–53], ubiquitination [54], S-nitrosylation [55], and O-GlcNAcylation [56]. The phosphorylation of Ser-637 by protein kinase A (PKA) [51, 53], calcium (Ca^2+^)/calmodulin-dependent protein kinase (CaM Kinase) [57], and Proto-oncogene serine/threonine-protein kinase Pim-1 (Pim1) [58] has been shown to prevent the mitochondrial translocation of Drp1. In contrast, the phosphorylation of Ser-616 by Cdk1/cyclin B (a key mitotic kinase) has been reported to promote mitochondrial fission by Drp1 during mitosis [59, 60]. Under conditions of high cytosolic Ca^2+^, dephosphorylation of Drp1 at Ser-637 by calcineurin induces mitochondrial fission [51, 52, 61–63]. The ablation of calcineurin in skeletal muscle leads to Ser-637 hyperphosphorylation of Drp1 causing elongation of mitochondria into ‘power-cable’-shaped filaments and increased mitochondrial respiration [64]. In hyperglycemic conditions, O-GlcNAcylation of OPA1 [65] and Drp1 [56] causes dephosphorylation of Ser-637 and the translocation of Drp1 to the OMM.

More recently, it has also been demonstrated that AMPK can phosphorylate Mff to trigger mitochondrial fission in response to stress thereby initiating mitophagy of the mitochondrial fragments that have extensive damage [66]. Knockdown of the OMM-associated E3 ubiquitin ligase MARCH5 was found to selectively inhibit ubiquitination and proteasomal degradation of MiD49 leading to mitochondrial fission, a phenotype which was reversed by MARCH5 re-expression or MiD49 knockout [67].

### Mitochondrial Fission, Apoptosis and Necroptosis

Drp1 has been demonstrated to co-localize with BAX at the OMM in response to apoptotic stimuli [68]. Recent findings have also invoked Drp1-dependent mitochondrial fission via the involvement of MiD49/MiD51 as being pertinent for cristae remodeling during intrinsic apoptosis [30]. Stimulated emission depletion (STED) microscopy has shown that BAX assembles with BAK and Drp1 in the form of a ring to delineate the area required for mitochondrial outer membrane permeabilization (MOMP) [69]. The area enclosed by the BAX ring is devoid of mitochondrial outer membrane proteins such as Tom20, Tom22, and Sam50 [69]. Drp1 is also stabilized by SUMOylation to promote mitochondrial fragmentation, loss of MMP and cytochrome C release [70], although mitochondrial fragmentation does not necessarily equate to cell death [71, 72]. Inhibition of Drp1 slows down rather than fully inhibits apoptosis as other pro-apoptotic proteins may still be released from the mitochondria [63, 73]. The opposite holds true whereby Drp1-dependent mitochondrial fission can occur independently of BAX, BAK and Apoptotic protease activating factor 1 (APAF1) to amplify cell death caused by certain factors such as BID and oxidative stress [74]. Downstream of BAX/BAK, the activation of apoptosis triggers mitochondrial-anchored protein ligase (MAPL/MUL1)-dependent SUMOylation of Drp1, a process requisite for stabilization of ER/mitochondrial contact sites that act as hotspots for mitochondrial constriction, calcium flux, cristae remodeling, and cytochrome c release [75].

Another interesting observation with regard to the role of Drp1 lies in its effects on necroptosis – a pathway of programmed necrosis. Earlier studies suggested the plausible role of Drp1 in mediating mitochondrial fragmentation following recruitment by the RIP3-PGAM5 complex [76–79]. Later studies have however, refuted this claim [80, 81]. Using arenobufagin- and staurosporine -treated HCT116 cells – a human colon cell line, it was reported that PGAM5L, but not PGAM5S constitutes a prerequisite for the activation of BAX and dephosphorylation of Drp1 for intrinsic apoptosis execution [82].

### Mitochondrial Fission and Mitophagy

Mitochondrial fission is also essential for the degradation and removal of damaged mitochondria by mitophagy. Down-regulation of Drp1 induces mitochondrial elongation, impairs mitochondrial autophagy, and prompts mitochondrial dysfunction, leading to cardiac dysfunction and increased susceptibility to ischemia/reperfusion [83]. Nonetheless, the duration of Drp1 downregulation used in this study was a prolonged period (at least 72 h in cardiomyocytes; 4 weeks tamoxifen injection in Drp1-CKO mice; 12-week-old Drp1-hetCKO mice) [83], highlighting the detrimental effects of chronic inhibition of Drp1. Moreover, the PTEN-induced putative kinase (PINK1) serves as a pro-fission signal, independently of Parkin [84]. A reduced level of mitochondrial division still persists even in the absence of Drp1 and is negatively regulated by Parkin [85]. Under normal conditions, the scaffold protein A-kinase anchoring protein 1 (AKAP1) recruits protein kinase A (PKA) to the outer mitochondrial membrane to phosphorylate and inhibit Drp1. Following cellular damage, PINK1 triggers PKA displacement from AKAP1 to ensure fission of damaged mitochondria for organelle degradation [84].

## Mitochondrial Fusion Proteins

The fusion of 2 adjacent mitochondria to a single organelle requires the initial fusion of the OMM under the regulation of Mfn1 and Mfn2, followed by the fusion of the inner mitochondrial membrane (IMM) by OPA1 (reviewed in [86]). The mitofusins consist of a mitochondrial GTPase domain, a transmembrane domain, and a coiled-coil domain. The transmembrane domain functions to anchor these proteins to the mitochondrial membranes while the coiled-coil domains face the cytosol and mediate the formation of homotypic (Mfn1–Mfn1, Mfn2–Mfn2 and OPA1–OPA1) or heterotypic (Mfn1–Mfn2) physical connections between adjacent mitochondria [11, 87, 88].

### The Mitofusins Mfn1 and Mfn2

The GTPase activity of Mfn1 (which is higher than that for Mfn2 [89]) is regulated by the binding of G-protein β2 subunit to Mfn1 which decreases the motility of the latter and facilitates its clustering at specific foci on the OMM, thereby promoting the pro-fusion effects of Mfn1 [90]. Mfn2 expression is regulated by proteins linked to mitochondrial biogenesis such as PGC1-α and PGC1-β [91, 92]. The ubiquitination of Mfn1 and Mfn2 promotes the degradation of these proteins allowing unopposed mitochondrial fission during the selective removal of dysfunctional mitochondria by mitophagy [93, 94]. In addition, other components of the OMM may interact with Mfn1 and Mfn2 to modulate mitochondrial morphology, such as appoptosin, a mitochondrial carrier protein that is located in the IMM [95], and Smad2 which recruits the Rab and Ras Interactor 1 (RIN1) into a complex with Mfn2 to promote mitochondrial fusion [96]. More recent studies have unravelled the various pleiotropic non-fusion roles of the mitochondrial fusion proteins, which will be described in the following sections and summarised in Table 1.

**Table 1:** Experimental studies implicating pleiotropic non-fusion roles of the mitochondrial fusion proteins

Study	Condition	Cell type	Major non-fusion roles	Other findings
*Mfn1*				
[97]	oxidative stress (exposed to antimycin A (AMA))	MEFs and HeLa	Mfn1 is rapidly accumulated, inducing mitochondrial hyperfusion. Following that, MARCH5 binding to Mfn1 and its subsequent ubiquitylation of Mfn1 is significantly enhanced.	
*Mfn2*				
[98–105]	Genetic ablation of Mfn2	MEFs	Reduced contact between mitochondria-ER	Increased contact between mitochondria-ER [101–105]
[106–108]			Mfn2 co-localize with both BAX and BAK in the OMM, impairing the fusion	Increased mPTP formation via the combination of Mfn2, BAX and BAK leads to cell death
[9, 93, 94, 109, 110]	Loss of mitochondrial potential		Removal of Mfn2 via ubiquitination and proteosomal degradation inhibits its pro-fusion activity	Mitochondrial fission occurs leading to removal of the damaged mitochondrion by mitophagy
*Opa1*				
[113, 115]	Loss of mitochondrial potential	SH-SY5Y and MEFs	OPA1 is up-regulated by the NF-κB-responsive promoter elements following Parkin recruitment to maintain mitochondrial integrity and protect from cell death [113].	Stress also induces the metallopeptidase OMA1 to degrade the long isoforms of OPA1 and causes mitochondrial fragmentation for removal by mitophagy [115].
[120]	Apoptotic stimuli	MEFs	OPA1 prevents cytochrome c release to inhibit cell death by ‘stapling’ the cristae junctions closed [120]	
[122]		Mice ESCs	OPA1 also regulates formation and stability of respiratory chain supercomplexes (RCS) – components of the electron transport chain (ETC) arranged to facilitate transfer of electrons, via regulation of cristae morphology [122].	

### Mfn1 and Apoptotic Cell Death

Mitochondrial adaptation in mouse embryonic fibroblasts (MEFs) and HeLa cells subjected to oxidative stress (exposed to antimycin A (AMA), an inhibitor of electron transfer at complex III) has been shown to be driven by mitochondrial ubiquitin ligase membrane-associated RING-CH (MARCH5)-dependent quality control of acetylated Mfn1 [97]. In the presence of stress, Mfn1 is rapidly accumulated, inducing mitochondrial hyperfusion. Following that, MARCH5 binding to Mfn1 and its subsequent ubiquitylation of Mfn1 is significantly enhanced. The acetylation status of Mfn1 dictates the process of MARCH5 binding to Mfn1 and its ubiquitylation. Although yet to be shown in the heart, acceleration of Mfn1 degradation by MARCH5 under stress remains an important quality control system that inhibits mitochondrial aggregation and cell death [97].

### Pleiotropic Non-fusion Role of Mfn2 as an ER-Tethering Protein

Mfn2 also tethers the ER to the mitochondria for calcium signaling from the ER to mitochondria [8]. This tethering function allows formation of subcellular domains of high calcium concentration close to the mitochondrial calcium uniporter. In the heart, this allows calcium to be efficiently transferred from the sarcoplasmic reticulum (SR) to the mitochondria which is crucial for cardiac contractility [98]. The genetic ablation of Mfn2 in the heart disrupts the SR-mitochondrial tethering and causes an impairment of Ca^2+^ signaling, diminished mitochondrial respiratory function and deterioration in left ventricular (LV) systolic function [99]. Nevertheless, a quantitative electron microscopy (EM) analysis showed an increase in the number of close contacts between the two organelles, in Mfn2−/− MEF cells [100], a finding supported by multiple biochemical, morphological, functional, and genetic data from other studies in subsequent years [101–105]. Whether this discrepancy is cell-specific or reliant on the presence of other specific proteins at the ER-mitochondria interface remains to be further clarified. The advent of more advanced imaging techniques may shed further light on the function of Mfn2 as a tethering protein.

### Pleiotropic Non-fusion Role of Mfn2 in Apoptotic Cell Death

Apoptosis or cardiomyocyte cell death has been implicated in both acute and chronic heart diseases. The loss of myocardium constitutes an important pathogenic process in the heart and, hence, targeting the inhibition of apoptosis remains a viable therapeutic option. Mfn2 has been demonstrated to co-localize with both BAX and BAK in the OMM [68, 106]. BAX and BAK are pro-apoptotic proteins and the binding of BAX to Mfn2 impairs its pro-fusion capability [106]. Mfn2 also facilitates formation of the mitochondrial permeability transition pore (MPTP) and decreases stability of the mitochondrial membrane, thus promoting Drp1-mediated mitochondrial fission [107]. The absence of both BAX and BAK rescues the cells from MPTP opening and cell death by necrosis, yet mitochondrial fragmentation ensues [108]. Interestingly, genetic ablation of Mfn2 also prevented MPTP opening, thus suggesting that the combination of BAX-Mfn2 is sufficient to facilitate opening of the MPTP, which is achieved by forming hemifusion-related holes used in the exchange of ions during stress-induced MPTP opening [108]. These findings suggest that the BAX/BAK-Mfn2 interaction may be sufficient to promote MPTP formation and increase susceptibility to cell death. This notion was further supported using the BAX/BAK/cyclophilin D triple knockout mice where MI size was not substantially reduced when compared to the BAX/BAK double knockout mice [108].

### Pleiotropic Non-fusion Role of Mfn2 in Mitophagy

Recent data suggested that Mfn2 plays a pivotal role in the removal of damage mitochondria by mitophagy. Chen et al. [109] found that in damaged mitochondria with loss of mitochondrial membrane potential, Mfn2 is phosphorylated by PINK1 at Thr-111 and Ser-442. This process then facilitates binding of Parkin to the OMM which in turn ubiquitinates Mfn2 [9]. The ubiquitination of Mfn2 inhibits its pro-fusion activity allowing mitochondrial fission to take place, and also recruits p62 to the OMM resulting in the selective removal of the damaged mitochondria by mitophagy [9, 93, 94, 110]. Similarly, stress-induced JNK phosphorylation of Mfn2 leads to recruitment of the ubiquitin ligase (E3) Huwe1/Mule/ARF-BP1/HectH9/E3Histone/Lasu1 to Mfn2, with the BH3 domain of Huwe1 implicated in this interaction [109]. This results in ubiquitin-mediated proteasomal degradation of Mfn2, leading to mitochondrial fission and enhanced apoptotic cell death [109]. In addition, Wang et al. [10] have demonstrated that Mfn2 plays a crucial role in autophagy as a mediator of the of autophagosome-lysosome fusion, and deficiency of Mfn2 in the heart was found to result in a cardiomyopathy. Furthermore, age-induced reduction in Mfn2 levels in murine skeletal muscle has been reported to impair both mitophagy and mitochondrial quality, leading to an exacerbated age-related mitochondrial dysfunction [111]. A compensatory mechanism involving a ROS-dependent adaptive signaling pathway through induction of HIF-1α and BNIP3 is triggered as a result, raising the possibility of a similar feedback mechanism in the heart [111].

### OPA1

The activity of the IMM pro-fusion protein OPA1 is regulated by alternative splicing and post-translational modification (for a more detailed review please see [112]). In addition to its pro-fusion effects OPA1 has been demonstrated to display a number of pleiotropic non-fusion effects.

### Pleiotropic Non-fusion Role of OPA1 in Mitophagy

Parkin has been shown to be recruited to the linear ubiquitin assembly complex under conditions of stress that subsequently increases linear ubiquitination of NF-κB essential modulator (NEMO), which is essential for canonical NF-κB signaling. OPA1 is then up-regulated by the NF-κB-responsive promoter elements to maintain mitochondrial integrity and protect from cell death. The lack of mitophagy, however, did not hamper the Parkin-induced protection [113]. This finding however, has been confounded by another study which did not find a major role for Parkin in mediating OPA1 regulation [114]. Instead, the authors found that the protective effect of Parkin may rather be related to the ubiquitination of BAX and the limitation of its mitochondrial translocation to the mitochondria [114]. The authors also noted a role of the non-classical NF-κB pathway in the regulation of mitochondrial dynamics and OPA1 expression by showing that the absence of IKKα induces lower levels of OPA1 and a fragmented mitochondrial network [114]. Stress also induces the metallopeptidase OMA1 to degrade the long isoforms of OPA1 and causes mitochondrial fragmentation for removal by mitophagy [115]. Nevertheless, whether the loss of OPA1 per se will induce impairment of lysosome function in the heart, as can be seen in neurodegenerative models [116], remains to be clarified. Furthermore, in situ proximity ligation assay (PLA) in murine lung epithelial MLE-12 cells and human cervical adenocarcinoma HeLa cells showed that the hexameric intermembrane space protein, NDPK-D (or NM23-H4), binds cardiolipin and facilitates its redistribution to the OMM as a form of mitophagy-induction signal in association with OPA1 [117].

### Pleiotropic Non-fusion Role of OPA1 in Cristae Remodeling and Mitochondrial Apoptosis

The anti-apoptotic effects of OPA1 have been attributed to its role in regulating mitochondrial cristae morphology and cytochrome c distribution. The cristae or invaginations of the IMM can be remodeled into either a narrow/tight junction or a widened gap by tBID. This action is essential for redistribution of cytochrome c from the intra-cristal space into the intermembrane space (IMS) to initiate apoptosis [118–121]. OPA1, conversely, prevents cytochrome c release to inhibit cell death by ‘stapling’ these cristae junctions closed [120].

### Pleiotropic Non-fusion Role of OPA1 in Cristae Remodeling and Mitochondrial Respiratory Efficiency

OPA1 also regulates formation and stability of respiratory chain supercomplexes (RCS) – components of the electron transport chain (ETC) arranged to facilitate transfer of electrons, via regulation of cristae morphology [122]. This implicates OPA1 as a regulator of mitochondrial respiration and a potential target for regulating mitochondrial energy production. A genome-wide RNA interference (RNAi) screen identified reactive oxygen species modulator 1 (ROMO1) as a redox-regulated protein that is able to oligomerize OPA1 for mitochondrial fusion and maintenance of normal cristae morphology [123]. The presence of oxidative stress induces the formation of high-molecular weight ROMO1 complexes while knockdown of ROMO1 promoted mitochondrial fission and loss of cristae, causing impaired mitochondrial respiration and increased sensitivity to cell death stimuli [123]. To adapt to cellular metabolic demand, changes in energy substrate availability are sensed by mitochondrial SLC25A transporters, which in turn regulate OPA1 oligomerization [124]. OPA1 oligomerization modulates cristae width and regulates assembly of the ATP synthase, in a mitochondrial fusion-independent manner. A fusion-incompetent form of OPA1(Q297V) rescued OCR, ATP synthase assembly and cell growth of OPA1 KO MEFs in galactose media, which forces mitochondrial respiration for ATP production [124]. Data from T cells supports the link between mitochondrial morphology and metabolism whereby fusion in memory T (TM) cells configures electron transport chain (ETC) complex associations favoring oxidative phosphorylation (OXPHOS) and FAO, while fission in effector T (TE) cells leads to cristae expansion, reducing ETC efficiency and promoting aerobic glycolysis [125].

## Mitochondrial Morphology and the Adult Heart

**Fig. 2:**
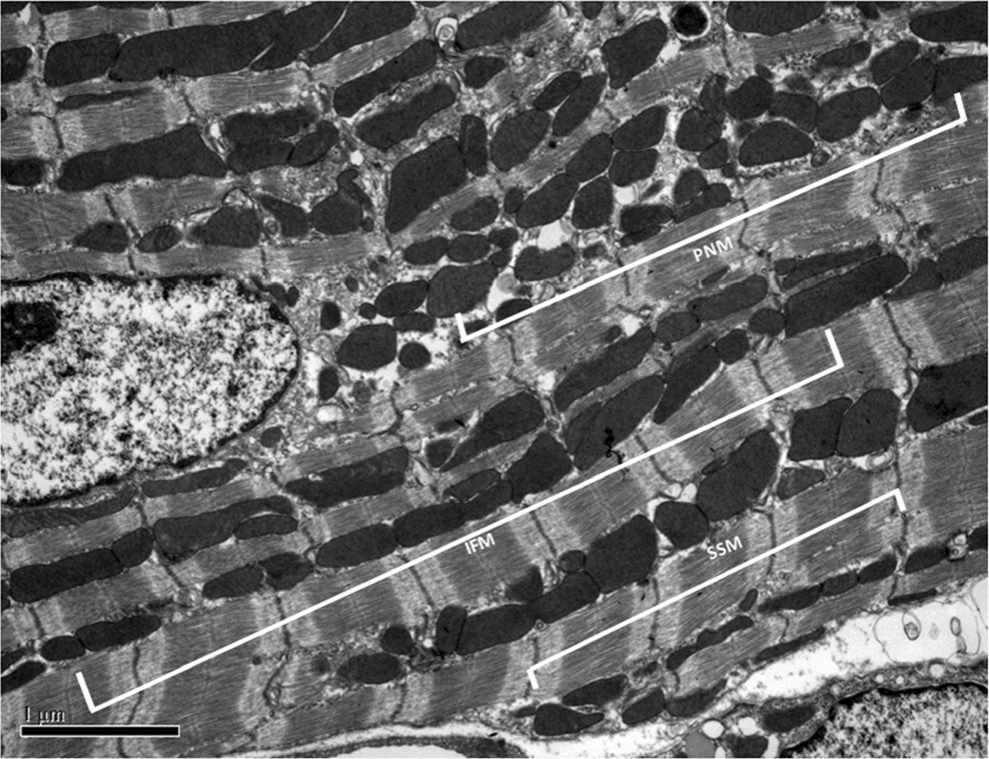
Electron microscopy image showing the 3 subpopulations of mitochondria in an adult cardiomyocyte. IFM, interfibrillar mitochondria; PNM, perinuclear mitochondria; SSM, subsarcolemmal mitochondria

Mitochondria in non-cardiac cells, cardiac cell lines, and neonatal cardiomyocytes are highly mobile and are distributed throughout the cytosol in a filamentous network. In contrast, in the adult cardiomyocyte, mitochondria are relatively static, constrained in their ability to move, are in close contact with each other, and are spatially arranged into 3 distinct sub-populations according to locality and function. The majority of the mitochondria are closely aligned adjacent to the myofibrils, with one or two mitochondria lying alongside each sarcomere – these have been termed the interfibrillar mitochondria (IFM), and are mainly involved in calcium signalling from the SR to mitochondria and providing the energy required for cardiomyocyte contraction [126, 127] (see Fig. 2). A smaller population of mitochondria are arranged in clusters beneath the subsarcolemmal membrane and are believed to provide the energy required for ion channel function and may be involved with cell signaling – termed subsarcolemmal mitochondria (SSM). The third subpopulation of mitochondria form clusters either side of the nucleus, and are termed perinuclear mitochondria (PNM), and are presumably required to provide energy for transcription [126, 127] (see Fig. 2).

Although relatively immobile due to this characteristic spatial arrangement, emerging data suggest that mitochondria in the adult heart can undergo mitochondrial fusion and fission (see reviews [3–5, 13, 128, 129]. The evidence for this has been provided by electron microscopy [130–134], and using confocal microscopy over a timescale ranging from seconds to minutes to follow the movement of fluorescent marker such as the photo-activatable mitochondrial green fluorescent protein [135, 136], mitochondrial red and green fluorescent proteins [137–140], Renilla luciferase complementation [141] to pH-sensitive fluorescent mitochondrial probes [142, 143] between mitochondria.

## Mitochondrial Fusion and Fission Proteins in Cardiac Health and Disease

### Cardiac Development and Growth

#### Mitochondrial Fission Proteins

During early cardiac differentiation, an upregulation of Drp1-mediated mitochondrial fission coincides with removal of mitochondria via SQSTM1 (sequestosome 1)-mediated mitophagy [144]. Mitochondria are then repopulated in the new myotube via PPARGC1A/PGC-1α (peroxisome proliferator-activated receptor gamma, coactivator 1 alpha)-mediated biogenesis, with a brisk upregulation of OPA1 levels [144]. Genetic ablation of Drp1 has been shown to be embryonically lethal at day E12.5 [145]. In the adult heart, ablation of Drp1 is associated with an impairment of mitophagy which leads to cardiomyopathy [83], accentuating the crucial role of Drp1-mediated fission in maintaining a healthy mitochondrial network. Judging from these findings, the inhibition of Drp1 as a therapeutic approach may not be as straightforward due to the fact that prolonged inhibition of Drp1 may be detrimental to normal cardiac function.

#### Mitochondrial Fusion Proteins

The importance of mitochondrial fusion in cardiac development has been delineated by death in utero during the mid-gestation period upon genetic ablation of both Mfn1 and Mfn2 [88]. Cardiac-specific ablation of the mitofusins in the embryo causes death at days E9.5–10.5 [146]. Kasahara et al. has demonstrated that the levels of Mfn2 and OPA1 play an essential role in cardiac development, with developmental arrest occurring at E13.5 when Mfn2 and OPA1 were absent. The observation postulates an association between mitochondrial dynamics and cardiac differentiation whereby calcium signaling linking calcineurin and Notch signalling was affected by the ablation of these pro-fusion proteins [147]. In the adult heart devoid of Mfn1, there appears to be a lack of distinct cardiac phenotype [148]. Conditional ablation of cardiac-specific Mfn2 (α-MHC-*Cre*), however, causes mitochondria to become pleomorphic and slightly enlarged while the LV exhibits modest hypertrophy with mild deterioration in systolic function [98, 99]. Mfn2-deficient MEFs exhibited a significantly increased respiration rate which correlated with increased levels of mitochondrial markers (TOM20 and NAO) as well as mitochondrial transcription factor A (TFAM) and peroxisome proliferator-activated receptor gamma coactivator 1-alpha (PGC-1α) protein levels while total ATP content remains unchanged [149]. The adaptive mechanism mediated by increased level of PGC-1α and TFAM transcription factor prevents an excessive depletion of mtDNA and severe impairment of cell metabolism [149]. Combined ablation of both Mfn1 and Mfn2 in the adult murine heart induces a lethal cardiomyopathy after several weeks, probably due to the mitochondrial fragmentation present in these hearts [146, 150].

Cardiac metabolism changes in line with the growth and maturation occurring in the first few days of post-natal growth. Mitochondrial respiration switches from glycolysis to oxidative phosphorylation during this period. Mitochondria also change shapes from a fragmented phenotype to an elongated shape aligned alongside the myofibrils, based on the increase in mitofusins [150] and reduction in cardiac hypoxia-inducible factor (HIF) signalling. This shift in HIF signaling also mediates an increase in fusion protein expression from E16.5 in a stepwise manner through to P10.5 with the largest increase taking place between P0.5 and P2.5 in MFN1 and MFN2 and between P2.5 and P10.5 in OPA1 while no changes were detectable in fission protein Drp1 and Fis1 levels over this time period [151]. Ablation of the mitofusins from the heart at the late embryonic stage causes severe mitochondrial dysfunction at 7 days (abnormal mitochondrial structure, down-regulated mitochondrial biogenesis genes, reduced mitochondrial DNA), development of cardiomyopathy, and all died before 14 days old [150]. The association between Mfn1 and PGC-1α during the postnatal growth of the heart lies in the ability of PGC-1α to stimulate the transcription of Mfn1 gene by co-activating the orphan nuclear receptor ERRα. Similarly, PGC-1α is crucial for a high-level expression of nuclear- and mitochondrial-encoded genes involved in mitochondrial energy transduction and OXPHOS pathways, and for full respiratory capacity [152], albeit dispensable for maintenance of mitochondria in the adult heart. Parkin-mediated mitophagy as a form of organelle replacement has been shown to be essential for normal perinatal cardiac mitochondrial and metabolic maturation [153]. Engineered mutant Mfn2 expressed from birth which cannot be PINK1-phosphorylated on Thr111 and Ser442 as required for Mfn2-Parkin binding disrupts developmental perinatal transformation of cardiac metabolism [153].

### Stem Cell Differentiation into Cardiomyocytes

Cardiac stem cells differentiating into adult cardiomyocytes experience a change in mitochondrial function and structure to accommodate for the switch from anaerobic glycolysis to oxidative phosphorylation, to support the increased metabolic demands of the differentiated beating cardiomyocyte ([154]; reviewed in [155]). In line with the change in mitochondrial metabolism, mitochondria change from a fragmented state (lacking cristae) in the embryonic stem cells (ESC), to an elongated state (with well-developed cristae), closely aligned with the myofibrils of the differentiated contractile cardiomyocyte [154]. This change in mitochondrial morphology is due to decreased Drp1 and increased Mfn2. The presence of the mitofusins constitutes a barrier to reprogramming whereby removing Mfn1/2 inhibits the p53-p21 pathway and promotes the conversion of somatic cells to a pluripotent state. The ablation of the mitofusins facilitates the glycolytic metabolic transition through the activation of the Ras-Raf and hypoxia-inducible factor 1α (HIF1α) signalling [156]. Interestingly, Kim et al. showed in the same year that Drp1 activation promoted differentiation of C2C12 myoblasts induced by serum starvation. Inhibiting mitochondrial fission in the same cell type via *mdivi-1* or Drp1K38A impairs myogenic maturation further supporting the role of both fission and fusion in cardiomyocyte differentiation [157]. A more recent study in *Drosophila* also demonstrated crosstalk between the fusion components (ChChd3, OPA1 and Marf) and the Hippo pathway in regulating cellular proliferation where a loss of fusion impairs proliferation during development [158]. The conflicting observations imply that the role of mitochondrial dynamics in cardiac differentiation is cell-specific and the cellular background (presence of certain mutations) may exert a more prominent role than expected. More recently, it was demonstrated that prohibitin 2 (PHB2) - a pleiotrophic factor mainly localised in mitochondria, is upregulated in undifferentiated mouse ES cells while differentiation causes the downregulation of the level of PHB2. This varying expression of PHB2 affects mitochondrial function via modulation of OPA1 processing, although the effects on OPA1 seem to be separate from the differentiation of ES cells [159].

It is interesting to note that reprogramming a somatic cell into induced pluripotent stem cells (iPSCs) requires an induction of genes involved in mitochondrial biogenesis (TFAM, NRF1), mitochondrial fusion (MFN1, MFN2) and glycine production while able to tolerate a certain degree of OXPHOS defects [160]. Reprogramming MEFs to iPSCs however, also induces a downregulation of the MAP kinase phosphatase Dusp6 to subsequently activate ERK signalling and promote a Drp1-dependent mitochondrial fission pathway that is necessary for pluripotency [161].

### Heart Failure

#### Mitochondrial Fission Proteins

A mutation in the Drp1 gene (C452F) in a mouse mutant, Python, caused an autosomal dominant form of dilated cardiomyopathy. The homozygous mutation was embryonically lethal but the heterozygous form achieved adulthood albeit it developed a severe dilated cardiomyopathy after 11 weeks. This cardiomyopathy was associated with reduced content of mitochondrial respiratory enzymes and ATP. In terms of mitochondrial morphology, mitochondria of heterozygous Python MEFs were abnormal with numerous long tubular mitochondria whilst homozygous Python MEFs had grossly abnormal mitochondria which appeared to be spherical and aggregated [162]. A further study by the same group refined the details by showing that the C452F mutation in mice not only increased Drp1 GTPase activity but also conferred resistance to oligomer disassembly, ultimately leading to impaired mitophagy, mitochondrial depolarization, aberrant calcium handling, impaired ATP synthesis, and activation of sterile myocardial inflammation [163]. Interestingly, a single human mutation in Drp1 (A395D), lying close to the C452F mutation in the middle domain of the protein was also previously described [164]. The mutant A395D protein is however defective for self-assembly and GTP hydrolysis [165]. Mitochondrial fragmentation evoked by enhanced Drp1-mediated fission in *Drosophila* cardiomyocytes did not adversely impact heart tube function [166]. Likewise, mitochondrial polarization status assessed by TMRE fluorescence was also not adversely impacted by Drp1-induced fragmentation [166]. The discrepancy in these observations for Drp1 mutations may be attributed to various factors, including: (1) species difference; (2) different Drp1 mutations (3) different gene promoters used for cardiomyocyte-specific gene manipulation.

In the SENP5-Tg (SENP5 (a SUMO isopeptidase) overexpressing) murine hearts, a decrease in SUMO attachment to Drp1 has been detected, leading to an enlargement of mitochondria at the early developmental stage. This was also accompanied by decreased cardiomyocyte proliferation and elevated apoptosis [167]. Excess mitochondrial fragmentation due to an upregulation of Drp1 contributes to impaired mitophagy and elevated mitochondrial ROS. Parkin is not abundant and has been found to be dispensable for constitutive mitophagy in normal hearts [168]. Removal of Drp1 by tamoxifen-inducible knockout in the adult mouse hearts, however, not only prevented mitochondrial fission, but also markedly upregulated Parkin, thus provoking mitophagic mitochondrial depletion that contributed to a lethal cardiomyopathy [168]. Concomitant conditional Parkin deletion with Drp1 ablation in adult mouse hearts prevented the upregulation of Parkin, while increasing 6-week survival, with an improved ventricular ejection fraction, mitigating adverse cardiac remodeling, and decreasing cardiac myocyte necrosis and replacement fibrosis [168]. Short-term observation of heart failure in mice subjected to transverse aortic constriction (TAC), however, showed that Drp1-associated mitochondrial autophagy is transiently activated and then downregulated in the mouse heart in response to pressure overload [169]. Downregulation of mitochondrial autophagy in this scenario plays an important role in mediating the development of mitochondrial dysfunction and heart failure, which can be reversed by injection of Tat-Beclin 1, a potent inducer of autophagy [169]. It has also been reported in murine hearts subjected to pressure overload that there is an aberrant activation of MMP-9 causing degradation of the gap junction protein connexin-43 (Cx-43) in the ventricular myocardium [170, 171]. The reduction in levels of Cx-43 was associated with increased fibrosis and ventricular dysfunction in heart failure [170]. Usage of the small molecule inhibitor of Drp1 – *mdivi-1*, normalized the decreased ratio of MFN2 to Drp1 [172] as well as the expression levels of MMP-9 and Cx-43, thus showing an improved cardiac function [171, 173, 174]. The intricate link between mitochondrial biogenesis and dynamics and mitophagy can also be seen from the use of Brg1 (Brahma-related gene 1) /Brm (Brahma) double mutant mice (cardiomyocyte-specific and inducible deletion of the SWI/SNF (SWItch/Sucrose Non-Fermentable) ATP-dependent chromatin remodeling complexes BRG1 and BRM in adult mice) [175]. These double-mutant mice exhibited increased mitochondrial biogenesis, increases in ‘mitophagy’, and late stage decrease in mitochondrial-shaping proteins that led to small, fragmented mitochondria following which a severe cardiomyopathy and death occur [175].

#### Mitochondrial Fusion Proteins

Cardiac ablation of both Mfn1 and Mfn2 causes a mild cardiomyopathy [98, 99]. Cardiac ablation of Mfn2 alone in the adult murine heart only manifests as increased sensitivity to acute IRI and development of late-onset LV dysfunction at 17 months of age. The underlying mechanisms associated with this include impaired autophagy, defective lipid metabolism, and decreased mitochondrial respiration (primarily at complex III) [10]. Silencing of MARF (mitochondrial assembly regulatory factor – orthologue of mammalian, Mitofusin) and OPA1 in the *Drosophila* fly heart tube leads to dilated cardiomyopathy, a phenomenon which could be rescued by over-expressing either of the human Mitofusins (MFN1 or MFN2) or superoxide dismutase 1, implicating impaired mitochondrial fusion and oxidative stress in the pathogenesis of the heart failure [176]. A further study conducted by the same group refined the details by demonstrating that scavenging ROS by overexpressing superoxide dismutase (SOD) or suppressing ROMO1 prevented mitochondrial and heart tube dysfunction provoked by OPA1 RNAi, but not by mitofusin/MARF RNAi. In contrast, enhancing the ability of endoplasmic/sarcoplasmic reticulum to handle stress by expressing Xbp1, an ER stress-responsive transcription factor, rescued the cardiomyopathy of mitofusin/MARF insufficiency without improving that caused by OPA1 deficiency [166]. This again reiterates, at least in *Drosophila* heart tubes, the fact that mitochondrial fission per se does not always lead to cardiac dysfunction. Rather, it is the co-occurrence of mitochondrial fission with enhanced ROS and ER stress that induces cardiomyopathy. Similarly, rat hearts followed for twelve to eighteen weeks after myocardial infarction exhibited a decrease in MFN2, an increase in Fis1, and no change in OPA1 expression [177]. The lack of Mfn2 also impairs Parkin-mediated mitophagy leading to an accumulation of damaged ROS-producing organelles and progressive heart failure [9, 178]. To circumvent this, suppressing mitochondrial ROS provides benefits in preventing depolarization of the mitochondria, respiratory impairment and structural degeneration. Nevertheless, it should be noted that suppression of mitochondrial ROS in these settings should be controlled as overly suppressing mitochondrial ROS was associated with impaired secondary autophagic pathways, suggesting the importance of mitochondrial ROS alongside mitochondrial dynamics in mediating mitophagy and minimize cardiac failure [179]. The lack of Parkin and ensuing disruption of mitophagy causes an accumulation of enlarged, hollow “donut” mitochondria with dilated cardiomyopathy [180]. Induction of mitochondrial fission prevented the cardiomyopathy and corrected mitochondrial dysfunction demonstrating the link among improper mitochondrial fusion, defective mitophagy and organ dysfunction [180].

Levels of OPA1 were also found to be decreased in a post-MI rat heart failure model and human dilated and ischemic cardiomyopathy tissue samples [181]. Heterozygous levels of OPA1 also reduced mitochondrial DNA copy number and decreased expression of nuclear antioxidant genes at 3–4 months [182]. Nevertheless, baseline cardiac function was found to be normal in OPA1-deficient mice, although cardiomyopathy associated with mitochondrial fragmentation and impaired mitochondrial function developed at 12 months of age [182]. The reason for the decline of OPA1 levels in heart failure requires further investigation. Similarly during initial compensatory cardiac hypertrophy, an increase in OPA1 and decrease in Drp1 occur in concert with decreased Parkin and Sirt1/AMPK-PGC-1α signalling, signifying a compromised mitochondrial remodelling system [183]. A recent study by Wai et al. demonstrated that dilated cardiomyopathy and heart failure was induced when cardiac-specific ablation of Yme1l in mice activated OMA1 and hastened OPA1 proteolysis, leading to mitochondrial fragmentation and altered cardiac metabolism [184]. This deleterious scenario was rescued by OMA1 deletion, which prevented OPA1 cleavage [184].

### Acute Ischemia-Reperfusion Injury and Cardioprotection

#### Mitochondrial Fission Proteins

Mitochondrial dysfunction induced by acute ischemia/reperfusion injury (IRI) is a critical determinant of cardiomyocyte death following acute myocardial infarction. In 2006, Brady et al. first made the observation that mitochondria undergo fission when HL-1 cardiac cells were subjected to simulated ischemia, generating fragmented mitochondria, a change in mitochondrial morphology which persisted into simulated reperfusion, and was reversed in the presence of pharmacological inhibition of p38 MAPK [185]. Two years later, Plotnikov et al. [186] noted that mitochondria in rat primary renal tubular epithelium and fibroblasts also underwent fragmentation during simulated IRI, and this effect was prevented by pre-treating the cells with either SkQ1 (a mitochondrial anti-oxidant), Li + (a non-specific GSK-3β inhibitor) or insulin. Whether mitochondrial fission induced by acute IRI was merely an epiphenomenon or a determinant of cell death in this setting, was investigated by our group, when we found that genetic inhibition of Drp1 in HL-1 cardiac cells (using a dominant-negative mutant of Drp1) [130], not only inhibited the mitochondrial fission induced by acute IRI but also reduced cell death in this setting, highlighting mitochondrial fission as a potential therapeutic target for cardioprotection. Our group went on to demonstrate that mitochondria in adult murine hearts also undergo fission in response to acute IRI, and that preventing this using *mdivi-1*, a small molecule inhibitor of Drp1, attenuated the death of adult cardiomyocytes subjected to simulated IRI and reduced in vivo MI size in the adult murine heart following acute IRI [130]. Subsequent studies have confirmed that adult cardiac mitochondria undergo fission in response to acute IRI, and that targeting Drp1 with dominant negative mutants [187], siRNA [188], *mdivi-1* [173, 189], P110 (a peptide inhibitor of the interaction between hFis and Drp1) [190], or Dynasore (a non-specific dynamin inhibitor) [191] reduced MI size in small animal models of acute IRI. Pharmacological inhibition of mitochondrial fission at the onset of reperfusion using *mdivi-1* [173], P110 [190] and most recently nanoparticle-mediated drug delivery of Mdivi1 (which was shown to be more effective than administering *mdivi-1* alone [192], has been shown in experimental studies to reduce MI size in small animal *ex* and in vivo models of acute IRI, demonstrating a clinically-relevant time-point for cardioprotection in AMI patients. However, a recent study in HL-1 cardiac cells failed to demonstrate any benefit with *mdivi-1* administered at the onset of reoxygenation, and actually showed an increase in cell death – the reason for which is unclear [193]. Whether therapeutic inhibition of mitochondrial fission can reduce MI size in a clinically-relevant large animal model remains to be determined. Given the potential non-specific effects of *mdivi-1* [194], novel, more -specific inhibitors of mitochondrial fission are needed to translate this therapeutic approach into the clinical setting. In this regard, the discovery of other components of the mitochondrial fission machinery such as ER-mediated mitochondrial pre-constriction and the Drp1 docking proteins in the OMM, Mff and MiD49/51, may provide novel therapeutic targets for inhibiting mitochondrial fission. It is important to appreciate that although acute inhibition of mitochondrial fission induced by acute IRI is cardioprotective, the chronic inhibition of Drp1 may be detrimental to the heart as the process of mitochondrial fission is necessary for the removal of damaged mitochondria by mitophagy [112, 196]. This was nicely illustrated by Ikeda et al. [83] who demonstrated that conditional cardiac-specific ablation of Drp1 induced mitochondrial elongation, suppressed mitophagy, increased MPTP opening susceptibility, resulting in a cardiomyopathy and increased MI size following acute IRI.

There are several unanswered questions concerning mitochondrial fission as a mediator of cell death in acute IRI, and as a target for cardioprotection. Firstly, the mechanism through which acute IRI induces mitochondrial fission is not clear - potential explanations include:

1. mitochondrial ROS [186, 189] which is generated during acute IRI is known to induce mitochondrial fission. Plotnikov et al. showed that pre-treatment of SkQ1 (a mitochondrial ROS scavenger) prevented the mitochondrial fission induced by simulated IRI [186], and Zaja et al. demonstrated that Trolox, a ROS scavenger, decreased pSer616 Drp1 levels and mitochondrial fission in HL-1 cardiac cells following simulated IRI [189].
2. cytosolic calcium overload which occurs during acute IRI may activate calcineurin thereby dephosphorylating and activating Drp1 [61, 195]. Hom et al. [196] demonstrated in both neonatal and adult cardiomyocyte that increasing cytosolic calcium concentration induced Drp1-dependent mitochondrial fission.

Secondly, the mechanism through which inhibiting mitochondrial fission induced by acute IRI protects the heart is not clear. We have shown that inhibiting mitochondrial fission may protect the heart against acute IRI by decreasing MPTP opening susceptibility [130], although the mechanisms linking mitochondrial fission to MPTP opening remain unclear and need to be further investigated.

A number of signaling pathways and/or cardioprotective interventions have been shown to inhibit Drp1-dependant mitochondrial fission induced by acute IRI:

1. Wang et al. [195] demonstrated that the p53-microRNA499-calcineurin-Drp1 axis operates in the heart to mediate mitochondrial fission and cardiomyocyte death in the setting of acute IRI in the heart, and therapeutic inhibition of this pathway was found to reduce MI size in murine hearts.
2. Using H9C2 myoblasts, Kim et al. [197] found that the mitochondrial scaffolding protein A-kinase anchoring protein 121 (AKAP121) inhibits mitochondrial fission following simulated IRI AKAP121 through PKA-dependent inhibitory phosphorylation of Drp1 and PKA-independent inhibition of Drp1-Fis1 interaction.
3. Pride et al. [198] have shown that nitrate-induce preconditioning protect H9C2 myoblasts against simulated IRI by activating PKA, phosphorylating Drp1 and inhibiting mitochondrial fission induced by acute IRI.
4. Kuzumic et al. [199] demonstrated that trimetazidine protected rat neonatal cardiomyocytes from palmitate-induced mitochondrial fission and dysfunction.
5. Zaja et al. [189] found that cyclin dependent kinase 1 (Cdk1) and protein kinase C isoform delta (PKC-δ) bind to and increased Drp1 activity, in the setting of simulated IRI in HL-1 cardiac cells.
6. Using an experimental model of isoproterenol (β-adrenergic receptor agonist)-induced ischemic injury in the rat heart, Xue et al. [200] demonstrated that vagal nerve stimulation attenuated the expression of the mitochondrial fission proteins (Drp1, hFis1), prevented mitochondrial fragmentation and inhibited MPTP opening, and decreased MI size, via the muscarinic-3 receptor/Ca2+/calmodulin-dependent kinase kinase β/AMPK signaling pathway.
7. DJ-1, also known as Park7 (Parkinson’s Disease autosomal recessive, early onset 7), is an evolutionarily conserved 189-amino acid protein, that has been shown to protect the brain and heart against acute IRI through its protective effects on mitochondrial function [201, 202]. Shimizu et al. [203] have demonstrated that DJ-1 reduces MI size by modifying the SUMOylation (a post-translational modification process in which small ubiquitin-like modifier proteins are covalently and reversibly conjugated to target proteins) of Drp1 and inhibiting mitochondrial fission in the murine heart [203].

#### Mitochondrial Fusion Proteins

The roles of the mitochondrial fusion proteins Mfn1, Mfn2 and OPA1 in susceptibility to acute IRI have been recently investigated in HL-1 cardiac cells, neonatal rodent cardiomyocytes, and adult rodent cardiomyocytes with both interesting and sometimes surprising results. The over-expression of Mfn2 in HL-1 cardiac cells was shown to induce mitochondrial elongation, lower MPTP opening susceptibility and reduce cell death following simulated acute IRI [130], and Mfn2 ablation in neonatal cardiomyocytes was shown to increase cell death following simulated IRI [99], findings which are consistent with the notion that mitochondrial fission induced by acute IRI is detrimental. However, in the adult heart, the genetic ablation of either cardiac-specific Mfn1 or Mfn2 was found to have opposing and unexpected effects with pleomorphic mitochondria, less MPTP opening and reduced cell death [107, 148, 204]. Similarly, although cardiac-specific ablation of both Mfn1 and Mfn2 (DKO) resulted in mitochondrial fragmentation, decreased respiratory function, and impaired myocardial contractile function [205], it resulted in less MPTP opening and reduced MI size following acute IRI. The reason for the unexpected results of ablating Mfn1 and/or Mfn2 in the heart are not known but may relate to pleiotropic effects of Mfn2. The absence of Mfn2 in the DKO mice increased the distance between SR and mitochondria and resulted in less mitochondrial calcium overload during acute IRI which may have, in part, contributed to the protected phenotype observed in the DKO mice [205]. These findings suggest that the acute inhibition of Mfn2 during acute IRI may provide a novel cardioprotective strategy and that the mechanism underlying this beneficial effect appears to be independent of its effects on mitochondrial morphology.

Partial ablation of OPA1 promotes formation of clusters of fused mitochondria and altered cristae with increased mitochondrial size. Sensitivity to MPTP opening however, was decreased [206]. OMA1 is activated in a BAX- and BAK-dependent fashion [207]. Activated OMA1 cleaved OPA1, an event that is critical for remodeling of mitochondrial cristae [207]. OMA1 knockdown in neuronal cells or renal proximal tubular cells prevented OPA1 proteolysis, fragmentation of mitochondria, cytochrome c release and cell death during ATP-depleted ischemia [208, 209]. The pro-survival kinase, Akt as well as its pharmacological activator, EPO has also been postulated to confer cardioprotection by means of elongating the mitochondria via modulation of Mfn1 [210], a finding similarly recapitulated by the pro-survival extracellular-signal-regulated kinase (ERK) [211]. Recent studies in the liver demonstrated a downregulation of SIRT1 following hepatic IRI, which was partially attributable to activation of calpains [212], while genetic overexpression or pharmacological activation of SIRT1 using resveratrol or SRT1720 markedly suppressed defective autophagy, onset of the mitochondrial permeability transition, and hepatocyte death after I/R [213]. The studies also identified Mfn2 as a novel target of SIRT1 in which SIRT1 deacetylates Mfn2 to mediate autophagy induction [212, 213]. Male adult rats exposed to four 5-min cycles of limb ischemia interspersed by 5 min of limb reperfusion, immediately prior to myocardial ischemia and 120 min of reperfusion (MI + RIPC group) experienced a smaller infarct size (−28%), increased mitochondrial fusion protein OPA1, and preserved mitochondrial morphology [214]. A mild overexpression of OPA1 also protected against cardiac ischemic injury measured in the form of reduced LDH release in 5 months old mice subjected to a Langendorff model of 40 min of ischemia followed by 15 min of reperfusion [215]. Whether this modest expression of the long form of OPA1 (∼10% in OPA1^tg^ hearts versus ∼0.6% of total OPA1 in WT) is able to reduce infarct size in an in vivo model of myocardial infarction remains to be investigated. The role of the mitochondrial-shaping proteins in susceptibility of the adult heart to acute IRI is complex and not easily determined, further complicated by the lack of an OPA1 or Mfn-specific activator.

### Left Ventricular Hypertrophy

#### Mitochondrial Fission Proteins

The development of left ventricular hypertrophy (LVH) has been linked to fragmentation of the mitochondria and enhanced mitophagy, due to an upregulation of Drp1 in a cell model of phenylephrine-induced cardiomyocyte hypertrophy [174, 177]. In order to prevent the development of LVH, Givvimani et al. employed *mdivi-1* to maintain the mitochondrial network and prompt the release of pro-angiogenic factors (CD31 and VEGF) while reducing collagen deposition [174], effects that were mirrored a year later in a quantitative phosphoproteomics study using myocardial samples at different time points following transverse aortic banding (TAB) [216]. The hypertrophic response and oxygen consumption were reduced in response to treatment with *mdivi-1* while the absence of *mdivi-1* saw phosphorylation of DRP1 S622 and subsequent mitochondrial translocation in TAB-treated mouse hearts and phenylephrine -treated rat neonatal cardiomyocytes [216]. These findings suggest a potential therapeutic strategy in acute inhibition of mitochondrial fission proteins to salvage LV hypertrophy.

#### Mitochondrial Fusion Proteins

MEK1/2–Erk1/2 expression is upregulated in LVH leading to a proliferation of VSMC, a phenomenon inhibited by Mfn2 (formerly known as hyperplasia suppressor gene or HSG) [217]. The same group used different experimental models of LVH (phenylephrine induced LVH in neonatal rat cardiomyocytes, spontaneously hypertensive rats, β2-adrenergic transgenic mice, and pressure overload LVH by transverse aortic constriction) to determine the downregulation of Mfn2 and upregulation of Erk1/2 [217]. Angiotensin-II treatment in neonatal rat myocytes similarly decreased expression of Mfn2 while elevating Akt levels. Over-expressing MFN2 reversed the angiotensin-ii-induced LVH in both neonatal cardiomyocytes and the intact rat heart [218]. In mice with cardiac-specific Mfn2 KO, the downregulation of Mfn2 impairs tethering of mitochondria to ER and subsequent Ca2+ signalling [9, 99]. Using the model of transverse aortic constriction (TAC), Piquereau et al. demonstrated that a partial deficiency in OPA1 also increases the susceptibility to LVH and cardiac dysfunction, albeit whether resuming the levels of OPA1 could rescue this phenotype is unknown [206].

### Anthracycline Cardiomyopathy

The aetiology of myocardial contractile dysfunction induced by anthracycline cardiotoxicity is unclear, although mitochondrial dysfunction and the production of ROS have been implicated. Marechal et al. [219] demonstrated the presence of mitochondrial fragmentation in hearts subjected to doxorubin, and this change in mitochondrial morphology could be prevented by the MPTP inhibitor, CsA. The importance of mitochondrial fission to the pathophysiology of doxorubicin cardiomyopathy was investigated by Gharanei et al. [172], who found that treatment with the Drp1 inhibitor, *mdivi-1*, attenuated mitochondrial fragmentation, prevented the cardiac dysfunction and increase in MI size induced by doxorubicin in the isolated rat heart, indicating the therapeutic potential for inhibiting mitochondrial fission as a strategy for preventing doxorubicin cardiomyopathy. Crucially, *mdivi-1* was found to have no effect on survival of a cancer cell line.

### Vascular Effects of Mitochondrial Fusion and Fission Proteins

#### Mitochondrial Fission Proteins

In native non-proliferative vascular smooth muscle cells (VSMC), the mitochondria are static and mainly ovoid in shape whereas during proliferation, the mitochondria become more mobile with varying shapes [220]. Angiotensin II stimulation of the cells activates Drp1 which will then interact with PKC-δ and subsequent MEK1/2–ERK1/2 signalling cascade and MMP2 [221]. Hyperproliferation of VSMCs also constitutes a pathogenic mechanism in diabetic vascular complications, associated with increased ROS leading to excessive Drp1- mediated mitochondrial fission [222]. Inhibiting Drp1 via *mdivi-1* impaired the proliferative response [221, 222]. Similarly, the presence of a positive feedback of ROS/mROS-DRP1 contributes to the suppression of pulmonary arterial smooth muscle cells (PASMC) apoptosis in hypoxic pulmonary vascular remodelling, a phenomenon circumvented by the use of ROS inhibitors such as N-acetylcysteine and mitochondrial-derived ROS inhibitor TEMPO [223]. The mechanism underlying pulmonary arterial hypertension (PAH) lies in the hyperproliferation of the PASMC, during which mitochondria divide to ensure equal re-distribution. Fragmentation of the mitochondria during hyperproliferation is due to an upregulation of Drp1 and downregulation of Mfn2, coupled with Cyclin B1/CDK1 phosphorylation of Drp1 at Ser-616. Reversal of PAH was achieved using the small molecule inhibitor of Drp1 – *mdivi-1* [60].

#### Mitochondrial Fusion Proteins

Originally known as a hyperplasia suppressor gene (HSG), Mfn2 functions to inhibit VSMC proliferation in different vasculo-proliferative conditions due to PKA-induced phosphorylation of Mfn2 at Ser442 [224, 225]. Over-expression of Mfn2 in an experimental animal model of angioplasty balloon-induced neointimal injury was found to inhibit VSMC proliferation, oxidized LDL and subsequent atheroma formation and carotid artery restenosis. Down-regulating Mfn2 conversely, enhanced VSMC proliferation, coupled with an increase in fatty acid oxidation and decrease in glucose oxidation. Conflicting data where down-regulation of Mfn2 in HeLa cells or T/G HA-VSMC cells suppressed proliferation has also been shown where interrupted autophagosome-lysosome fusion and impaired bioenergetics are the underlying factors [226].

During hypoxia-induced pulmonary hypertension in rats, downregulation of Mfn2 activates the PI3K/Akt pathway, thereby causing more cells to enter the S + G2/M phase of the cell cycle and inhibiting the mitochondrial apoptosis pathway [227]. These observations suggest that modifications in mitochondrial morphology and bioenergetics may underlie the hyperproliferative features of the VSMC, although the optimum balance of Mfn2 and associated mechanisms determine the endpoint of whether activation or suppression of proliferation occurs. In the settings of PAH, both Mfn2 and PGC-1alpha have been found to be down-regulated in PASMCs. Over-expression of Mfn2 reversed the phenotype of PAH. Similarly, both Mfn2 and PGC-1α were down-regulated in PASMC in two different experimental models of PAH, and in patients with PAH while Mfn2 reversed this phenotype. This seems to implicate Mfn2 as a novel therapeutic target for PAH [60], although whether preventing mitochondrial fission via the use of *mdivi-1* may be beneficial remain to be determined.

### Other Cardiac Conditions

Intriguingly, a recent experimental study by Sharp et al. [228] has demonstrated that pharmacological inhibition of Drp1 using *mdivi-1* improved time to return of spontaneous circulation and hemodynamics, thereby resulting in improved survival and neurological outcomes, in a murine model of cardiac arrest. Another interesting example is the work by Sumida et al. [229] where they showed that mouse kidney subjected to acute IRI induced mitochondrial fragmentation in the heart, resulting in apoptosis and cardiac dysfunction, the effects of which could be reverse by pharmacological inhibition of Drp1 using *mdivi-1*.

### Diabetes

In obesity and type 2 diabetes (DM), the loss of mitochondrial function in white adipose tissue is associated with a reduction in whole body insulin sensitivity. It has been demonstrated in the kidneys of streptozotocin-induced diabetic mice that there is an upregulation of translocase of inner mitochondrial membrane 44 (Timm44) [230]. Timm44 anchors mitochondrial heat-shock protein 70 to the translocase of inner mitochondrial membrane 23 complex and facilitates the import of mitochondria-targeted pre-proteins into the mitochondrial matrix. Over-expression of Timm44 in mice fed high-fat/high-sucrose chow protects from type 2 diabetes and obesity. Adipocyte size and pro-fission proteins were reduced while the pro-fusion proteins were induced when the mice were fed standard chow [230]. The discrepancy behind these varying levels of mitochondrial-shaping proteins in these mice when fed different diets remains unexplained although mitochondria ultimately became elongated.

Mitochondrial biogenesis remains unperturbed in the diabetic myocardium, in accordance with the absence of a difference in citrate synthase activities and PGC1-α protein expression [231]. Electron microscopy imaging of right atrial tissue sections also showed no difference in interfibrillar mitochondrial density between diabetic and nondiabetic patients [231]. However, a shift toward smaller cardiac mitochondria was observed in DM with a significantly lower mean mitochondrial length (1.14 μm) in diabetic versus (1.41 μm) in nondiabetic patients, due to a lower expression level of Mfn1 [231]. Interestingly, the content of myocardial MFN1 protein correlated negatively with HbA1C concentrations. Autophagy levels in the diabetic myocardium remained normal as well, albeit Atg5 was significantly decreased [231]. These observations established the association of mitochondrial dysfunction (mitochondrial fragmentation with decreased Mfn1) with decreased contractile performance in cardiac muscle of diabetic patients before the onset of clinical cardiomyopathy. Obesity, conversely, was not associated with any major perturbation of mitochondrial function [231], although a study conducted in the same year using high-fat diet -induced obese mice showed the opposite [232]. A recent study also showcased the Drp1 monitoring of the mitochondrial network which is important for glucose-stimulated insulin secretion in pancreatic beta cells [233]. The optimum balance between fusion and fission may, thus, govern the proper function of the pancreatic beta cells and susceptibility to diabetes.

The presence of diabetes has been reported to render the heart resistant to cardioprotective strategies such as ischemic preconditioning and postconditioning. Yu et al. [234] found that hyperglycemia blocked the cardioprotective effect elicited of sevoflurane postconditioning by inducing mitochondrial fission in neonatal rat cardiomyocytes, an effect which was reversed using the Drp1 inhibitor, *mdivi-1*.

## Therapeutic Targeting of Mitochondrial Fusion and Fission Proteins

A number of strategies have been investigated to modulate mitochondrial morphology including: genetic approaches such as micro-RNAs [235]; upregulation of heme oxygenase-1 (HO-1) to mediate mitochondrial quality control [236]; restoration of Connexin43 networks [237]; and even exercise to upregulate mitochondrial biogenesis factors [238]. Remote ischemic preconditioning (RIPC) has been shown to induce upregulation of OPA1 [214], and sevoflurane postconditioning has been reported to suppress the decline of OPA1 and increase in f Drp1 and Parkin induced by IRI [234].

Pharmacological interventions to target mitochondrial morphology have included the use of melatonin [239, 240], *Tribulus terrestris L*. fruit methanol extract [241], mitochondria-targeted molecules MitoQ and SS31 that have been shown to upregulate the pro-fusion proteins while downregulating the pro-fission proteins as well as inducing the mitochondrial biogenesis genes PGC1α, PGC1β, Nrf1, Nrf2 and TFAM [242], coenzyme Q10 to rescue loss of Mfn2-associated respiratory impairment [243], resveratrol or SRT1720 to induce SIRT1 [213] or even metformin to inhibit ROS-associated mitochondrial fission by upregulating Drp1 phosphorylation (Ser 637) in an AMPK-dependent manner, and then suppressing ER stress [244]. The GLP-1 peptide-mimetic exenatide which has been used for the treatment of type 2 diabetes prevents neointimal layer formation in response to endothelial damage and atherosclerotic lesion formation in aortic tissue. Torres et al. have shown that GLP-1 induced a Ser-637 phosphorylation in the mitochondrial fission protein Drp1, and decreased Drp1 mitochondrial localization, thereby inhibiting VSMC migration and proliferation [245].

**Fig. 3:**
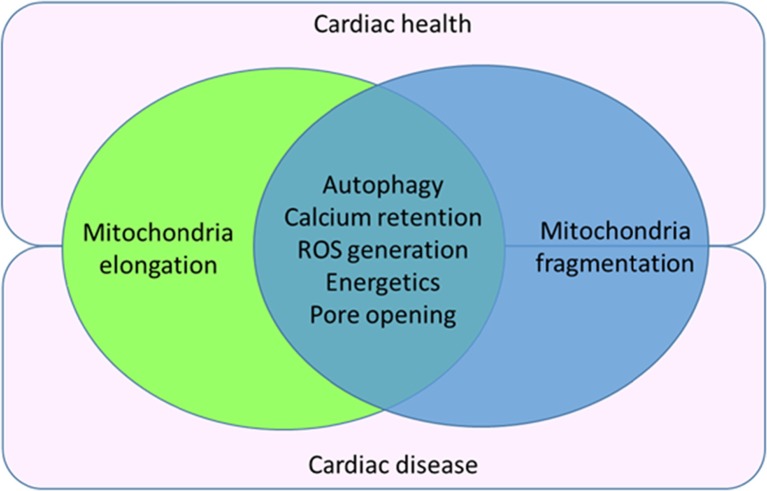
Image showing the co-dependency of both mitochondrial fusion and fission in cardiac health and disease. Changes in mitochondrial morphology in the form of fusion and fission can affect autophagy, calcium signaling, ROS generation, mitochondrial energetics and MPTP opening, all of which can impact on cardiac health and disease

Drugs that have been documented to protect the heart by inhibiting the MPTP such as cyclosporine-A may well serve to modulate mitochondrial morphology, although this needs to be investigated. Similarly, although well-known mitochondrial morphology modulators such as *mdivi-1* have been shown to protect the heart against acute IRI, their potential off-target effects and efficacy in a large animal model and humans remains to be tested. The optimum time to modulate mitochondrial morphology during the course of cardiac dysfunction progression to achieve a beneficial effect is not easy to determine. The drug delivery vehicle which may affect the potency of the drug is another crucial factor to be taken into account. In addition, one should realize that the hope of conferring cardioprotection by simply modulating mitochondrial morphology towards a defined phenotype may not be beneficial in the long-term given that it will interfere with mitochondrial homeostasis by disturbing the balance of mitochondrial fusion and fission (see Fig. 3) [246].

## Conclusions

Emerging studies suggest that changes in mitochondrial morphology occur in different cardiac disease and pharmacological targeting of the mitochondrial fusion and fission proteins may provide a novel therapeutic strategy for treating cardiac diseases such as acute myocardial infarction, LVH, PAH, and heart failure. However, it is important to take into consideration the pleiotropic roles of the mitochondrial shaping proteins especially the fusion proteins, Mfn2 and OPA1, when targeting the mitochondrial shaping proteins as a treatment for cardiac disease. Furthermore, although acute inhibition or activation of the shaping proteins may be beneficial in certain cardiac diseases, the chronic inhibition or activation of either mitochondrial fusion or fission proteins may have detrimental off-target effects.

## Abbreviations

AKAP1: protein A-kinase anchoring protein 1
AMA: antimycin A
AMPK: 5′ AMP-activated protein kinase
APAF1: Apoptotic protease activating factor 1
Ca^2+^: calcium
CaM Kinase: calmodulin-dependent protein kinase
Cdk1: cyclin dependent kinase 1
Drp1: dynamin-related protein 1
ER: endoplasmic reticulum
ERK: extracellular-signal-regulated kinase
ESC: embryonic stem cells
ETC: electron transport chain
HFpEF: heart failure with preserved ejection fraction
hFis1: human fission protein-1
HIF: hypoxia-inducible factor
IFM: interfibrillar mitochondria
IMM: inner mitochondrial membrane
INF2: inverted formin-2
iPSCs: induced pluripotent stem cells
IRI: ischemia-reperfusion injury
LV: left ventricular
LVH: left ventricular hypertrophy
MAPL: mitochondrial-anchored protein ligase
MARCH5: membrane-associated RING-CH
MARF: mitochondrial assembly regulatory factor
Mcl-1: myeloid cell leukemia factor 1
Mff: mitochondrial fission factor
Mfn1 / 2: Mitofusins 1 and 2
MI: myocardial infarction
MiD49/51: mitochondrial dynamics proteins of 49 (MiD49) and 51 kDa (MiD51)
MPTP: mitochondrial permeability transition pore
mtDNA: mitochondrial DNA
NEMO: NF-κB essential modulator
OMM: outer mitochondrial membrane
OPA1: Optic Atrophy 1
PAH: pulmonary arterial hypertension
PASMC: pulmonary arterial smooth muscle cells
PGC-1α: peroxisome proliferator-activated receptor gamma coactivator 1-alpha
Pim1: Proto-oncogene serine/threonine-protein kinase Pim-1
PINK1: PTEN-induced putative kinase
PLA: proximity ligation assay
PNM: perinuclear mitochondria
RCS: respiratory chain supercomplexes
RIN1: Rab and Ras Interactor 1
RIPC: remote ischemic preconditioning
SOD: superoxide dismutase
SR: sarcoplasmic reticulum
SSM: subsarcolemmal mitochondria
STED: Stimulated emission depletion
SUMO: Small Ubiquitin-like Modifier
TAB: transverse aortic banding
Tfam: mitochondrial transcription factor A
Timm44: translocase of inner mitochondrial membrane 44
VSMC: vascular smooth muscle cells
XIAP: X-linked inhibitor of apoptosis protein

## References

[1] Chen H, Vermulst M, Wang YE (2010). Mitochondrial fusion is required for mtDNA stability in skeletal muscle and tolerance of mtDNA mutations. Cell..

[2] Glancy B, Hartnell LM, Malide D (2015). Mitochondrial reticulum for cellular energy distribution in muscle. Nature..

[3] Ong S-B, Hall AR, Hausenloy DJ (2013). Mitochondrial dynamics in cardiovascular health and disease. Antioxid Redox Signal..

[4] Ong S-B, Gustafsson AB (2012). New roles for mitochondria in cell death in the reperfused myocardium. Cardiovasc Res..

[5] Ong S-B, Hausenloy DJ (2010). Mitochondrial morphology and cardiovascular disease. Cardiovasc Res..

[6] Ong S-B, Kalkhoran SB, Cabrera-Fuentes HA, Hausenloy DJ (2015). Mitochondrial fusion and fission proteins as novel therapeutic targets for treating cardiovascular disease. Eur J Pharmacol.

[7] Merkwirth C, Langer T (2008). Mitofusin 2 builds a bridge between ER and mitochondria. Cell..

[8] De Brito OM, Scorrano L (2008). Mitofusin 2 tethers endoplasmic reticulum to mitochondria. Nature..

[9] Chen Y, Dorn GW (2013). PINK1-phosphorylated mitofusin 2 is a Parkin receptor for culling damaged mitochondria. Science..

[10] Zhao T, Huang X, Han L (2012). Central role of mitofusin 2 in autophagosome-lysosome fusion in cardiomyocytes. J Biol Chem..

[11] Chan DC (2006). Mitochondrial fusion and fission in mammals. Ann Rev Cell Dev Biol..

[12] Chen H, Chan DC. Emerging functions of mammalian mitochondrial fusion and fission. Hum Mol Genet. 2005;14 Spec No:R283–9. 10.1093/hmg/ddi270 16244327

[13] Piquereau J, Caffin F, Novotova M (2013). Mitochondrial dynamics in the adult cardiomyocytes: which roles for a highly specialized cell?. Front Physiol..

[14] Kuznetsov AV, Hermann M, Saks V, Hengster P, Margreiter R (2009). The cell-type specificity of mitochondrial dynamics. Int J Biochem Cell Biol..

[15] McBride HM, Neuspiel M, Wasiak S (2006). Mitochondria: more than just a powerhouse. Curr Biol..

[16] Chan DC (2006). Mitochondria: dynamic organelles in disease, aging, and development. Cell..

[17] Ingerman E, Perkins EM, Marino M (2005). Dnm1 forms spirals that are structurally tailored to fit mitochondria. J Cell Biol..

[18] Legesse-Miller A, Massol RH, Kirchhausen T (2003). Constriction and Dnm1p recruitment are distinct processes in mitochondrial fission. Mol Biol Cell..

[19] Smirnova E, Shurland DL, Ryazantsev SN, van der Bliek AM (1998). A human dynamin-related protein controls the distribution of mitochondria. J Cell Biol..

[20] Smirnova E, Griparic L, Shurland DL, van der Bliek AM (2001). Dynamin-related protein Drp1 is required for mitochondrial division in mammalian cells. Mol Biol Cell..

[21] Losón OC, Song Z, Chen H, Chan DC (2013). Fis1, Mff, MiD49, and MiD51 mediate Drp1 recruitment in mitochondrial fission. Mol Biol Cell..

[22] Osellame LD, Singh AP, Stroud DA (2016). Cooperative and independent roles of the Drp1 adaptors Mff, MiD49 and MiD51 in mitochondrial fission. J Cell Sci..

[23] Liu R, Chan DC (2015). The mitochondrial fission receptor Mff selectively recruits oligomerized Drp1. Mol Biol Cell..

[24] Otera H, Wang C, Cleland MM (2010). Mff is an essential factor for mitochondrial recruitment of Drp1 during mitochondrial fission in mammalian cells. J Cell Biol..

[25] Gandre-Babbe S, van der Bliek AM (2008). The novel tail-anchored membrane protein Mff controls mitochondrial and peroxisomal fission in mammalian cells. Mol Biol Cell..

[26] Palmer CS, Elgass KD, Parton RG, Osellame LD, Stojanovski D, Ryan MT (2013). Adaptor proteins MiD49 and MiD51 can act independently of Mff and Fis1 in Drp1 recruitment and are specific for mitochondrial fission. J Biol Chem..

[27] Losόn OC, Meng S, Ngo H, Liu R, Kaiser JT, Chan DC (2015). Crystal structure and functional analysis of MiD49, a receptor for the mitochondrial fission protein Drp1. Protein Sci..

[28] Richter V, Palmer CS, Osellame LD (2014). Structural and functional analysis of MiD51, a dynamin receptor required for mitochondrial fission. J Cell Biol..

[29] Palmer CS, Osellame LD, Laine D, Koutsopoulos OS, Frazier AE, Ryan MT (2011). MiD49 and MiD51, new components of the mitochondrial fission machinery. EMBO Rep..

[30] Otera H, Miyata N, Kuge O, Mihara K (2016). Drp1-dependent mitochondrial fission via MiD49/51 is essential for apoptotic cristae remodeling. J Cell Biol..

[31] Samangouei P, Elder J, Burke N, Hall A, Hausenloy D. P143Targeting the mitochondrial fission proteins, MiD49 and MiD51, as a therapeutic strategy for cardioprotection. Cardiovasc Res. 2014;103 Suppl :S25.

[32] Stojanovski D, Koutsopoulos OS, Okamoto K, Ryan MT (2004). Levels of human Fis1 at the mitochondrial outer membrane regulate mitochondrial morphology. J Cell Sci..

[33] James DI, Parone PA, Mattenberger Y, Martinou J-C (2003). hFis1, a novel component of the mammalian mitochondrial fission machinery. J Biol Chem..

[34] Zhu P-P, Patterson A, Stadler J, Seeburg DP, Sheng M, Blackstone C (2004). Intra- and intermolecular domain interactions of the C-terminal GTPase effector domain of the multimeric dynamin-like GTPase Drp1. J Biol Chem..

[35] Koirala S, Guo Q, Kalia R (2013). Interchangeable adaptors regulate mitochondrial dynamin assembly for membrane scission. Proc Natl Acad Sci U S A..

[36] Macdonald PJ, Stepanyants N, Mehrotra N (2014). A dimeric equilibrium intermediate nucleates Drp1 reassembly on mitochondrial membranes for fission. Mol Biol Cell..

[37] Macdonald PJ, Francy CA, Stepanyants N (2016). Distinct splice variants of dynamin-related protein 1 differentially utilize mitochondrial fission factor as an effector of cooperative GTPase activity. J Biol Chem..

[38] Clinton RW, Francy CA, Ramachandran R, Qi X, Mears JA (2016). Dynamin-related protein 1 oligomerization in solution impairs functional interactions with membrane-anchored mitochondrial fission factor. J Biol Chem..

[39] Ban-Ishihara R, Tomohiro-Takamiya S, Tani M, Baudier J, Ishihara N, Kuge O (2015). COX assembly factor ccdc56 regulates mitochondrial morphology by affecting mitochondrial recruitment of Drp1. FEBS Lett.

[40] Wu W, Lin C, Wu K (2016). FUNDC1 regulates mitochondrial dynamics at the ER-mitochondrial contact site under hypoxic conditions. EMBO J..

[41] Wu W, Li W, Chen H, Jiang L, Zhu R, Feng D. FUNDC1 is a novel mitochondrial-associated-membrane (MAM) protein required for hypoxia-induced mitochondrial fission and mitophagy. Autophagy. 2016;1–2 10.1080/15548627.2016.1193656 PMC5082786 27314574

[42] Chen M, Chen Z, Wang Y (2016). Mitophagy receptor FUNDC1 regulates mitochondrial dynamics and mitophagy. Autophagy..

[43] Morciano G, Giorgi C, Balestra D (2016). Mcl-1 involvement in mitochondrial dynamics is associated with apoptotic cell death. Mol Biol Cell..

[44] Korobova F, Ramabhadran V, Higgs HN (2013). An actin-dependent step in mitochondrial fission mediated by the ER-associated formin INF2. Science..

[45] De Vos KJ, Allan VJ, Grierson AJ, Sheetz MP (2005). Mitochondrial function and actin regulate dynamin-related protein 1-dependent mitochondrial fission. Curr Biol..

[46] Friedman JR, Lackner LL, West M, DiBenedetto JR, Nunnari J, Voeltz GK (2011). ER tubules mark sites of mitochondrial division. Science..

[47] Pagliuso A, Tham TN, Stevens JK (2016). A role for septin 2 in Drp1-mediated mitochondrial fission. EMBO Rep..

[48] Li S, Xu S, Roelofs BA (2015). Transient assembly of F-actin on the outer mitochondrial membrane contributes to mitochondrial fission. J Cell Biol..

[49] Ji W, Hatch AL, Merrill RA, Strack S, Higgs HN (2015). Actin filaments target the oligomeric maturation of the dynamin GTPase Drp1 to mitochondrial fission sites. Elife..

[50] Figueroa-Romero C, Iñiguez-Lluhí JA, Stadler J (2009). SUMOylation of the mitochondrial fission protein Drp1 occurs at multiple nonconsensus sites within the B domain and is linked to its activity cycle. FASEB J..

[51] Cribbs JT, Strack S (2007). Reversible phosphorylation of Drp1 by cyclic AMP-dependent protein kinase and calcineurin regulates mitochondrial fission and cell death. EMBO Rep..

[52] Cho S-G, Du Q, Huang S, Dong Z (2010). Drp1 dephosphorylation in ATP depletion-induced mitochondrial injury and tubular cell apoptosis. Am J Physiol Renal Physiol..

[53] Chang C-R, Blackstone C (2007). Cyclic AMP-dependent protein kinase phosphorylation of Drp1 regulates its GTPase activity and mitochondrial morphology. J Biol Chem..

[54] Nakamura N, Kimura Y, Tokuda M, Honda S, Hirose S (2006). MARCH-V is a novel mitofusin 2- and Drp1-binding protein able to change mitochondrial morphology. EMBO Rep..

[55] Cho D-H, Nakamura T, Fang J (2009). S-nitrosylation of Drp1 mediates beta-amyloid-related mitochondrial fission and neuronal injury. Science..

[56] Gawlowski T, Suarez J, Scott B (2012). Modulation of dynamin-related protein 1 (DRP1) function by increased O-linked-β-N-acetylglucosamine modification (O-GlcNAc) in cardiac myocytes. J Biol Chem..

[57] Han X-J, Lu Y-F, Li S-A (2008). CaM kinase I alpha-induced phosphorylation of Drp1 regulates mitochondrial morphology. J Cell Biol..

[58] Din S, Mason M, Völkers M (2013). Pim-1 preserves mitochondrial morphology by inhibiting dynamin-related protein 1 translocation. Proc Natl Acad Sci U S A..

[59] Taguchi N, Ishihara N, Jofuku A, Oka T, Mihara K (2007). Mitotic phosphorylation of dynamin-related GTPase Drp1 participates in mitochondrial fission. J Biol Chem..

[60] Marsboom G, Toth PT, Ryan JJ (2012). Dynamin-related protein 1-mediated mitochondrial mitotic fission permits hyperproliferation of vascular smooth muscle cells and offers a novel therapeutic target in pulmonary hypertension. Circ Res..

[61] Cereghetti GM, Stangherlin A, Martins de Brito O (2008). Dephosphorylation by calcineurin regulates translocation of Drp1 to mitochondria. Proc Natl Acad Sci U S A..

[62] Sandebring A, Thomas KJ, Beilina A (2009). Mitochondrial alterations in PINK1 deficient cells are influenced by calcineurin-dependent dephosphorylation of dynamin-related protein 1. PLoS One..

[63] Estaquier J, Arnoult D (2007). Inhibiting Drp1-mediated mitochondrial fission selectively prevents the release of cytochrome c during apoptosis. Cell Death Differ..

[64] Pfluger PT, Kabra DG, Aichler M (2015). Calcineurin Links Mitochondrial Elongation with Energy Metabolism. Cell Metab..

[65] Makino A, Suarez J, Gawlowski T (2011). Regulation of mitochondrial morphology and function by O-GlcNAcylation in neonatal cardiac myocytes. Am J Physiol Regul Integr Comp Physiol..

[66] Toyama EQ, Herzig S, Courchet J (2016). AMP-activated protein kinase mediates mitochondrial fission in response to energy stress. Science..

[67] Xu S, Cherok E, Das S (2016). Mitochondrial E3 ubiquitin ligase MARCH5 controls mitochondrial fission and cell sensitivity to stress-induced apoptosis through regulation of MiD49 protein. Mol Biol Cell..

[68] Karbowski M, Lee Y-J, Gaume B (2002). Spatial and temporal association of Bax with mitochondrial fission sites, Drp1, and Mfn2 during apoptosis. J Cell Biol..

[69] Große L, Wurm CA, Brüser C, Neumann D, Jans DC, Jakobs S (2016). Bax assembles into large ring-like structures remodeling the mitochondrial outer membrane in apoptosis. EMBO J..

[70] Wasiak S, Zunino R, McBride HM (2007). Bax/Bak promote sumoylation of DRP1 and its stable association with mitochondria during apoptotic cell death. J Cell Biol..

[71] Parone PA, James DI, Da Cruz S (2006). Inhibiting the mitochondrial fission machinery does not prevent Bax/Bak-dependent apoptosis. Mol Cell Biol..

[72] Wakabayashi J, Zhang Z, Wakabayashi N (2009). The dynamin-related GTPase Drp1 is required for embryonic and brain development in mice. J Cell Biol..

[73] Frank S, Gaume B, Bergmann-Leitner ES (2001). The role of dynamin-related protein 1, a mediator of mitochondrial fission, in apoptosis. Dev Cell..

[74] Oettinghaus B, D’Alonzo D, Barbieri E (2016). DRP1-dependent apoptotic mitochondrial fission occurs independently of BAX, BAK and APAF1 to amplify cell death by BID and oxidative stress. Biochim Biophys Acta - Bioenerg..

[75] Prudent J, Zunino R, Sugiura A, Mattie S, Shore GC, McBride HM (2015). MAPL SUMOylation of Drp1 Stabilizes an ER/Mitochondrial Platform Required for Cell Death. Mol Cell..

[76] Vanlangenakker N, Vanden Berghe T, Krysko DV, Festjens N, Vandenabeele P (2008). Molecular mechanisms and pathophysiology of necrotic cell death. Curr Mol Med..

[77] Cho YS, Challa S, Moquin D (2009). Phosphorylation-driven assembly of the RIP1-RIP3 complex regulates programmed necrosis and virus-idoi nduced inflammation. Cell..

[78] Wang Z, Jiang H, Chen S, Du F, Wang X (2012). The mitochondrial phosphatase PGAM5 functions at the convergence point of multiple necrotic death pathways. Cell..

[79] Chen W, Zhou Z, Li L (2013). Diverse sequence determinants control human and mouse receptor interacting protein 3 (RIP3) and mixed lineage kinase domain-like (MLKL) interaction in necroptotic signaling. J Biol Chem..

[80] Moujalled DM, Cook WD, Murphy JM, Vaux DL (2014). Necroptosis induced by RIPK3 requires MLKL but not Drp1. Cell Death Dis..

[81] Remijsen Q, Goossens V, Grootjans S (2014). Depletion of RIPK3 or MLKL blocks TNF-driven necroptosis and switches towards a delayed RIPK1 kinase-dependent apoptosis. Cell Death Dis..

[82] Xu W, Jing L, Wang Q (2015). Bax-PGAM5L-Drp1 complex is required for intrinsic apoptosis execution. Oncotarget..

[83] Ikeda Y, Shirakabe A, Maejima Y (2015). Endogenous Drp1 mediates mitochondrial autophagy and protects the heart against energy stress. Circ Res..

[84] Pryde KR, Smith HL, Chau K-Y, Schapira AHV (2016). PINK1 disables the anti-fission machinery to segregate damaged mitochondria for mitophagy. J Cell Biol..

[85] Roy M, Itoh K, Iijima M, Sesaki H (2016). Parkin suppresses Drp1-independent mitochondrial division. Biochem Biophys Res Commun..

[86] Youle RJ, van der Bliek AM (2012). Mitochondrial fission, fusion, and stress. Science..

[87] Koshiba T, Detmer SA, Kaiser JT, Chen H, McCaffery JM, Chan DC (2004). Structural basis of mitochondrial tethering by mitofusin complexes. Science..

[88] Chen H, Detmer SA, Ewald AJ, Griffin EE, Fraser SE, Chan DC (2003). Mitofusins Mfn1 and Mfn2 coordinately regulate mitochondrial fusion and are essential for embryonic development. J Cell Biol..

[89] Ishihara N, Eura Y, Mihara K (2004). Mitofusin 1 and 2 play distinct roles in mitochondrial fusion reactions via GTPase activity. J Cell Sci..

[90] Zhang J, Liu W, Liu J (2010). G-protein β2 subunit interacts with mitofusin 1 to regulate mitochondrial fusion. Nat Commun..

[91] Zorzano A, Liesa M, Palacín M (2009). Mitochondrial dynamics as a bridge between mitochondrial dysfunction and insulin resistance. Arch Physiol Biochem..

[92] Liesa M, Borda-d’Agua B, Medina-Gómez G (2008). Mitochondrial fusion is increased by the nuclear coactivator PGC-1beta. PLoS One..

[93] Tanaka A, Cleland MM, Xu S (2010). Proteasome and p97 mediate mitophagy and degradation of mitofusins induced by Parkin. J Cell Biol..

[94] Gegg ME, Cooper JM, Chau K-Y, Rojo M, Schapira AHV, Taanman J-W (2010). Mitofusin 1 and mitofusin 2 are ubiquitinated in a PINK1/parkin-dependent manner upon induction of mitophagy. Hum Mol Genet..

[95] Zhang C, Shi Z, Zhang L (2016). Appoptosin interacts with mitochondrial outer-membrane fusion proteins and regulates mitochondrial morphology. J Cell Sci..

[96] Kumar S, Pan CC, Shah N (2016). Activation of mitofusin2 by smad2-RIN1 complex during mitochondrial fusion. Mol Cell..

[97] Park Y-Y, Nguyen OTK, Kang H, Cho H (2014). MARCH5-mediated quality control on acetylated Mfn1 facilitates mitochondrial homeostasis and cell survival. Cell Death Dis..

[98] Chen Y, Csordás G, Jowdy C (2012). Mitofusin 2-containing mitochondrial-reticular microdomains direct rapid cardiomyocyte bioenergetic responses via interorganelle Ca(2+) crosstalk. Circ Res..

[99] Papanicolaou KN, Khairallah RJ, Ngoh GA (2011). Mitofusin-2 maintains mitochondrial structure and contributes to stress-induced permeability transition in cardiac myocytes. Mol Cell Biol..

[100] Cosson P, Marchetti A, Ravazzola M, Orci L (2012). Mitofusin-2 independent juxtaposition of endoplasmic reticulum and mitochondria: an ultrastructural study. PLoS One..

[101] Filadi R, Greotti E, Turacchio G, Luini A, Pozzan T, Pizzo P (2015). Mitofusin 2 ablation increases endoplasmic reticulum-mitochondria coupling. Proc Natl Acad Sci U S A..

[102] Li L, Gao G, Shankar J, Joshi B, Foster LJ, Nabi IR (2015). p38 MAP kinase-dependent phosphorylation of the Gp78 E3 ubiquitin ligase controls ER-mitochondria association and mitochondria motility. Mol Biol Cell..

[103] Wang PTC, Garcin PO, Fu M (2015). Distinct mechanisms controlling rough and smooth endoplasmic reticulum contacts with mitochondria. J Cell Sci..

[104] Filadi R, Greotti E, Turacchio G, Luini A, Pozzan T, Pizzo P (2016). Presenilin 2 modulates endoplasmic reticulum-mitochondria coupling by tuning the antagonistic effect of mitofusin 2. Cell Rep..

[105] Leal NS, Schreiner B, Pinho CM (2016). Mitofusin-2 knockdown increases ER-mitochondria contact and decreases amyloid β-peptide production. J Cell Mol Med..

[106] Neuspiel M, Zunino R, Gangaraju S, Rippstein P, McBride H (2005). Activated mitofusin 2 signals mitochondrial fusion, interferes with Bax activation, and reduces susceptibility to radical induced depolarization. J Biol Chem..

[107] Papanicolaou KN, Phillippo MM, Walsh K (2012). Mitofusins and the mitochondrial permeability transition: the potential downside of mitochondrial fusion. Am J Physiol Heart Circ Physiol..

[108] Whelan RS, Konstantinidis K, Wei A-C (2012). Bax regulates primary necrosis through mitochondrial dynamics. Proc Natl Acad Sci U S A..

[109] Leboucher GP, Tsai YC, Yang M (2012). Stress-induced phosphorylation and proteasomal degradation of mitofusin 2 facilitates mitochondrial fragmentation and apoptosis. Mol Cell..

[110] Glauser L, Sonnay S, Stafa K, Moore DJ (2011). Parkin promotes the ubiquitination and degradation of the mitochondrial fusion factor mitofusin 1. J Neurochem..

[111] Sebastián D, Sorianello E, Segalés J (2016). Mfn2 deficiency links age-related sarcopenia and impaired autophagy to activation of an adaptive mitophagy pathway. EMBO J..

[112] Burke N, Hall AR, Hausenloy DJ (2015). OPA1 in cardiovascular health and disease. Curr Drug Targets..

[113] Müller-Rischart AK, Pilsl A, Beaudette P (2013). The E3 ligase parkin maintains mitochondrial integrity by increasing linear ubiquitination of NEMO. Mol Cell..

[114] Laforge M, Rodrigues V, Silvestre R (2016). NF-κB pathway controls mitochondrial dynamics. Cell Death Differ..

[115] Baker MJ, Lampe PA, Stojanovski D (2014). Stress-induced OMA1 activation and autocatalytic turnover regulate OPA1-dependent mitochondrial dynamics. EMBO J..

[116] Demers-Lamarche J, Guillebaud G, Tlili M (2016). Loss of mitochondrial function impairs lysosomes. J Biol Chem..

[117] Kagan VE, Jiang J, Huang Z (2016). NDPK-D (NM23-H4)-mediated externalization of cardiolipin enables elimination of depolarized mitochondria by mitophagy. Cell Death Differ..

[118] Scorrano L, Ashiya M, Buttle K (2002). A distinct pathway remodels mitochondrial cristae and mobilizes cytochrome c during apoptosis. Dev Cell..

[119] Kim T-H, Zhao Y, Ding W-X (2004). Bid-cardiolipin interaction at mitochondrial contact site contributes to mitochondrial cristae reorganization and cytochrome C release. Mol Biol Cell..

[120] Frezza C, Cipolat S (2006). Martins de Brito O, et al OPA1 controls apoptotic cristae remodeling independently from mitochondrial fusion. Cell..

[121] Epand RF, Martinou J-C, Fornallaz-Mulhauser M, Hughes DW, Epand RM (2002). The apoptotic protein tBid promotes leakage by altering membrane curvature. J Biol Chem..

[122] Cogliati S, Frezza C, Soriano ME (2013). Mitochondrial cristae shape determines respiratory chain supercomplexes assembly and respiratory efficiency. Cell..

[123] Norton M, Ng AC-H, Baird S et al. (2014) ROMO1 is an essential redox-dependent regulator of mitochondrial dynamics. Sci Signal 7:ra10 10.1126/scisignal.2004374 24473195

[124] Patten DA, Wong J, Khacho M (2014). OPA1-dependent cristae modulation is essential for cellular adaptation to metabolic demand. EMBO J..

[125] Buck MD, O’Sullivan D, Klein Geltink RI (2016). Mitochondrial dynamics controls T cell fate through metabolic programming. Cell..

[126] Hoppel CL, Tandler B, Fujioka H, Riva A (2009). Dynamic organization of mitochondria in human heart and in myocardial disease. Int J Biochem Cell Biol..

[127] Amchenkova AA, Bakeeva LE, Chentsov YS, Skulachev VP, Zorov DB (1988). Coupling membranes as energy-transmitting cables. I. Filamentous mitochondria in fibroblasts and mitochondrial clusters in cardiomyocytes. J Cell Biol..

[128] Chen H, Chan DC (2004). Mitochondrial dynamics in mammals. Curr Top Dev Biol..

[129] Braschi E, McBride HM (2010). Mitochondria and the culture of the Borg: understanding the integration of mitochondrial function within the reticulum, the cell, and the organism. Bioessays..

[130] Ong S-B, Subrayan S, Lim SY, Yellon DM, Davidson SM, Hausenloy DJ (2010). Inhibiting mitochondrial fission protects the heart against ischemia/reperfusion injury. Circulation..

[131] Shim S-H, Xia C, Zhong G (2012). Super-resolution fluorescence imaging of organelles in live cells with photoswitchable membrane probes. Proc Natl Acad Sci U S A..

[132] Brunstein M, Wicker K, Hérault K, Heintzmann R, Oheim M (2013). Full-field dual-color 100-nm super-resolution imaging reveals organization and dynamics of mitochondrial and ER networks. Opt Express..

[133] Sherman S, Nachmias D, Elia N (2015). A simple, straightforward correlative live-cell-imaging-structured-illumination-microscopy approach for studying organelle dynamics. Microsc Res Tech..

[134] Lo CY-W, Chen S, Creed SJ (2016). Novel super-resolution capable mitochondrial probe, MitoRed AIE, enables assessment of real-time molecular mitochondrial dynamics. Sci Rep..

[135] Twig G, Graf SA, Wikstrom JD, et al. Tagging and tracking individual networks within a complex mitochondrial web with photoactivatable GFP. Am J Physiol Cell Physiol. 2006;291:C176–84. 10.1152/ajpcell.00348.2005 16481372

[136] Magrané J, Cortez C, Gan W-B, Manfredi G (2014). Abnormal mitochondrial transport and morphology are common pathological denominators in SOD1 and TDP43 ALS mouse models. Hum Mol Genet..

[137] Legros F, Lombès A, Frachon P, Rojo M (2002). Mitochondrial fusion in human cells is efficient, requires the inner membrane potential, and is mediated by mitofusins. Mol Biol Cell..

[138] Hernandez G, Thornton C, Stotland A (2013). MitoTimer: a novel tool for monitoring mitochondrial turnover. Autophagy..

[139] Ferree AW, Trudeau K, Zik E (2013). MitoTimer probe reveals the impact of autophagy, fusion, and motility on subcellular distribution of young and old mitochondrial protein and on relative mitochondrial protein age. Autophagy..

[140] Huang S, Han R, Zhuang Q (2015). New photostable naphthalimide-based fluorescent probe for mitochondrial imaging and tracking. Biosens Bioelectron..

[141] Huang H, Choi S-Y, Frohman MA (2010). A quantitative assay for mitochondrial fusion using Renilla luciferase complementation. Mitochondrion..

[142] McWilliams TG, Prescott AR, Allen GFG (2016). Mito-QC illuminates mitophagy and mitochondrial architecture in vivo. J Cell Biol..

[143] Lee MH, Park N, Yi C (2014). Mitochondria-immobilized pH-sensitive off-on fluorescent probe. J Am Chem Soc..

[144] Sin J, Andres AM, Taylor DJR (2016). Mitophagy is required for mitochondrial biogenesis and myogenic differentiation of C2C12 myoblasts. Autophagy..

[145] Manczak M, Sesaki H, Kageyama Y, Reddy PH (2012). Dynamin-related protein 1 heterozygote knockout mice do not have synaptic and mitochondrial deficiencies. Biochim Biophys Acta..

[146] Chen Y, Liu Y, Dorn GW (2011). Mitochondrial fusion is essential for organelle function and cardiac homeostasis. Circ Res..

[147] Kasahara A, Cipolat S, Chen Y, Dorn GW, Scorrano L (2013). Mitochondrial fusion directs cardiomyocyte differentiation via calcineurin and Notch signaling. Science..

[148] Papanicolaou KN, Ngoh GA, Dabkowski ER (2012). Cardiomyocyte deletion of mitofusin-1 leads to mitochondrial fragmentation and improves tolerance to ROS-induced mitochondrial dysfunction and cell death. Am J Physiol Heart Circ Physiol..

[149] Kawalec M, Boratyńska-Jasińska A, Beręsewicz M, Dymkowska D, Zabłocki K, Zabłocka B. Mitofusin 2 deficiency affects energy metabolism and mitochondrial biogenesis in MEF cells. PLoS One. 2015;10:e0134162. 10.1371/journal.pone.0134162 PMC4521854 26230519

[150] Papanicolaou KN, Kikuchi R, Ngoh GA (2012). Mitofusins 1 and 2 are essential for postnatal metabolic remodeling in heart. Circ Res..

[151] Neary MT, Ng K-E, Ludtmann MHR (2014). Hypoxia signaling controls postnatal changes in cardiac mitochondrial morphology and function. J Mol Cell Cardiol..

[152] Martin OJ, Lai L, Soundarapandian MM (2014). A role for peroxisome proliferator-activated receptor γ coactivator-1 in the control of mitochondrial dynamics during postnatal cardiac growth. Circ Res..

[153] Gong G, Song M, Csordas G, Kelly DP, Matkovich SJ, Dorn GW (2015). Parkin-mediated mitophagy directs perinatal cardiac metabolic maturation in mice. Science..

[154] Chung S, Dzeja PP, Faustino RS, Perez-Terzic C, Behfar A, Terzic A (2007). Mitochondrial oxidative metabolism is required for the cardiac differentiation of stem cells. Nat Clin Pract Cardiovasc Med..

[155] Rehman J (2010). Empowering self-renewal and differentiation: the role of mitochondria in stem cells. J Mol Med (Berl)..

[156] Son MJ, Kwon Y, Son M-Y (2015). Mitofusins deficiency elicits mitochondrial metabolic reprogramming to pluripotency. Cell Death Differ..

[157] Kim B, Kim J-S, Yoon Y, Santiago MC, Brown MD, Park J-Y (2013). Inhibition of Drp1-dependent mitochondrial division impairs myogenic differentiation. Am J Physiol Regul Integr Comp Physiol..

[158] Deng Q, Guo T, Zhou X, Xi Y, Yang X, Ge W (2016). Crosstalk between mitochondrial fusion and the hippo pathway in controlling cell proliferation during drosophila development. Genetics..

[159] Kowno M, Watanabe-Susaki K, Ishimine H (2014). Prohibitin 2 regulates the proliferation and lineage-specific differentiation of mouse embryonic stem cells in mitochondria. PLoS One..

[160] Hung SSC, Van Bergen NJ, Jackson S (2016). Study of mitochondrial respiratory defects on reprogramming to human induced pluripotent stem cells. Aging..

[161] Prieto J, León M, Ponsoda X (2016). Early ERK1/2 activation promotes DRP1-dependent mitochondrial fission necessary for cell reprogramming. Nat Commun..

[162] Ashrafian H, Docherty L, Leo V (2010). A mutation in the mitochondrial fission gene Dnm1l leads to cardiomyopathy. PLoS Genet..

[163] Cahill TJ, Leo V, Kelly M (2015). Resistance of dynamin-related protein 1 oligomers to disassembly impairs mitophagy, resulting in myocardial inflammation and heart failure. J Biol Chem..

[164] Waterham HR, Koster J, van Roermund CWT, Mooyer PAW, Wanders RJA, Leonard JV (2007). A lethal defect of mitochondrial and peroxisomal fission. N Engl J Med..

[165] Chang C-R, Manlandro CM, Arnoult D (2010). A lethal de novo mutation in the middle domain of the dynamin-related GTPase Drp1 impairs higher order assembly and mitochondrial division. J Biol Chem..

[166] Bhandari P, Song M, Dorn GW (2015). Dissociation of mitochondrial from sarcoplasmic reticular stress in Drosophila cardiomyopathy induced by molecularly distinct mitochondrial fusion defects. J Mol Cell Cardiol..

[167] Kim EY, Zhang Y, Beketaev I (2015). SENP5, a SUMO isopeptidase, induces apoptosis and cardiomyopathy. J Mol Cell Cardiol..

[168] Song M, Gong G, Burelle Y (2015). Interdependence of parkin-mediated mitophagy and mitochondrial fission in adult mouse hearts. Circ Res..

[169] Shirakabe A, Zhai P, Ikeda Y (2016). Drp1-dependent mitochondrial autophagy plays a protective role against pressure overload-induced mitochondrial dysfunction and heart failure. Circulation..

[170] Jansen JA, van Veen TAB, de Jong S (2012). Reduced Cx43 expression triggers increased fibrosis due to enhanced fibroblast activity. Circ Arrhythm Electrophysiol..

[171] Givvimani S, Tyagi N, Sen U (2010). MMP-2/TIMP-2/TIMP-4 versus MMP-9/TIMP-3 in transition from compensatory hypertrophy and angiogenesis to decompensatory heart failure. Arch Physiol Biochem..

[172] Gharanei M, Hussain A, Janneh O, Maddock H (2013). Attenuation of doxorubicin-induced cardiotoxicity by mdivi-1: a mitochondrial division/mitophagy inhibitor. PLoS One..

[173] Sharp WW, Fang YH, Han M (2014). Dynamin-related protein 1 (Drp1)-mediated diastolic dysfunction in myocardial ischemia-reperfusion injury: therapeutic benefits of Drp1 inhibition to reduce mitochondrial fission. FASEB J..

[174] Givvimani S, Munjal C, Tyagi N, Sen U, Metreveli N, Tyagi SC (2012). Mitochondrial division/mitophagy inhibitor (Mdivi) ameliorates pressure overload induced heart failure. PLoS One..

[175] Bultman SJ, Holley DW, de Ridder G (2016). BRG1 and BRM SWI/SNF ATPases redundantly maintain cardiomyocyte homeostasis by regulating cardiomyocyte mitophagy and mitochondrial dynamics in vivo. Cardiovasc Pathol..

[176] Dorn GW, Clark CF, Eschenbacher WH (2011). MARF and Opa1 control mitochondrial and cardiac function in Drosophila. Circ Res..

[177] Javadov S, Rajapurohitam V, Kilić A (2011). Expression of mitochondrial fusion-fission proteins during post-infarction remodeling: the effect of NHE-1 inhibition. Basic Res Cardiol..

[178] Bhandari P, Song M, Chen Y, Burelle Y, Dorn GW (2014). Mitochondrial contagion induced by Parkin deficiency in Drosophila hearts and its containment by suppressing mitofusin. Circ Res..

[179] Song M, Chen Y, Gong G, Murphy E, Rabinovitch PS, Dorn GW (2014). Super-suppression of mitochondrial reactive oxygen species signaling impairs compensatory autophagy in primary mitophagic cardiomyopathy. Circ Res..

[180] Bhandari P, Song M, Chen Y, Burelle Y, Dorn GW (2014). Mitochondrial contagion induced by Parkin deficiency in Drosophila hearts and its containment by suppressing mitofusin. Circ Res..

[181] Chen L, Gong Q, Stice JP, Knowlton AA (2009). Mitochondrial OPA1, apoptosis, and heart failure. Cardiovasc Res..

[182] Chen L, Liu T, Tran A (2012). OPA1 mutation and late-onset cardiomyopathy: mitochondrial dysfunction and mtDNA instability. J Am Heart Assoc..

[183] Tang Y, Mi C, Liu J, Gao F, Long J (2014). Compromised mitochondrial remodeling in compensatory hypertrophied myocardium of spontaneously hypertensive rat. Cardiovasc Pathol..

[184] Wai T, García-Prieto J, Baker MJ (2015). Imbalanced OPA1 processing and mitochondrial fragmentation cause heart failure in mice. Science.

[185] Brady NR, Hamacher-Brady A, Gottlieb RA (2006). Proapoptotic BCL-2 family members and mitochondrial dysfunction during ischemia/reperfusion injury, a study employing cardiac HL-1 cells and GFP biosensors. Biochim Biophys Acta..

[186] Plotnikov EY, Vasileva AK, Arkhangelskaya AA, Pevzner IB, Skulachev VP, Zorov DB (2008). Interrelations of mitochondrial fragmentation and cell death under ischemia/reoxygenation and UV-irradiation: protective effects of SkQ1, lithium ions and insulin. FEBS Lett..

[187] Zepeda R, Kuzmicic J, Parra V (2014). Drp1 loss-of-function reduces cardiomyocyte oxygen dependence protecting the heart from ischemia-reperfusion injury. J Cardiovasc Pharmacol..

[188] Wang P, Wang P, Liu B (2015). Dynamin-related protein Drp1 is required for Bax translocation to mitochondria in response to irradiation-induced apoptosis. Oncotarget..

[189] Zaja I, Bai X, Liu Y (2014). Cdk1, PKCδ and calcineurin-mediated Drp1 pathway contributes to mitochondrial fission-induced cardiomyocyte death. Biochem Biophys Res Commun..

[190] Disatnik M-H, Ferreira JCB, Campos JC (2013). Acute inhibition of excessive mitochondrial fission after myocardial infarction prevents long-term cardiac dysfunction. J Am Heart Assoc..

[191] Gao D, Zhang L, Dhillon R, Hong T-T, Shaw RM, Zhu J (2013). Dynasore protects mitochondria and improves cardiac lusitropy in Langendorff perfused mouse heart. PLoS One..

[192] Ishikita A, Matoba T, Ikeda G (2016). Nanoparticle-mediated delivery of mitochondrial division inhibitor 1 to the myocardium protects the heart from ischemia-reperfusion injury through inhibition of mitochondria outer membrane permeabilization: a new therapeutic modality for acute myocardial. J Am Hear Assoc..

[193] Dong Y, Undyala VVR, Przyklenk K (2016). Inhibition of mitochondrial fission as a molecular target for cardioprotection: critical importance of the timing of treatment. Basic Res Cardiol..

[194] So EC, Hsing C-H, Liang C-H, Wu S-N (2012). The actions of mdivi-1, an inhibitor of mitochondrial fission, on rapidly activating delayed-rectifier K+ current and membrane potential in HL-1 murine atrial cardiomyocytes. Eur J Pharmacol..

[195] Wang J-X, Jiao J-Q, Li Q (2011). miR-499 regulates mitochondrial dynamics by targeting calcineurin and dynamin-related protein-1. Nat Med..

[196] Hom JR, Gewandter JS, Michael L, Sheu S-S, Yoon Y (2007). Thapsigargin induces biphasic fragmentation of mitochondria through calcium-mediated mitochondrial fission and apoptosis. J Cell Physiol..

[197] Kim H, Scimia MC, Wilkinson D (2011). Fine-tuning of Drp1/Fis1 availability by AKAP121/Siah2 regulates mitochondrial adaptation to hypoxia. Mol Cell..

[198] Kamga Pride C, Mo L, Quesnelle K (2014). Nitrite activates protein kinase A in normoxia to mediate mitochondrial fusion and tolerance to ischaemia/reperfusion. Cardiovasc Res..

[199] Kuzmicic J, Parra V, Verdejo HE (2014). Trimetazidine prevents palmitate-induced mitochondrial fission and dysfunction in cultured cardiomyocytes. Biochem Pharmacol..

[200] Xue R-Q, Sun L, Yu X-J, Li D-L, Zang W-J (2017). Vagal nerve stimulation improves mitochondrial dynamics via an M3 receptor/CaMKKβ/AMPK pathway in isoproterenol-induced myocardial ischaemia. J Cell Mol Med..

[201] Dongworth RK, Mukherjee UA, Hall AR (2014). DJ-1 protects against cell death following acute cardiac ischemia-reperfusion injury. Cell Death Dis..

[202] Mukherjee UA, Ong S-B, Ong S-G, Hausenloy DJ (2015). Parkinson’s disease proteins: Novel mitochondrial targets for cardioprotection. Pharmacol Ther..

[203] Shimizu Y, Lambert JP, Nicholson CK (2016). DJ-1 protects the heart against ischemia–reperfusion injury by regulating mitochondrial fission. J Mol Cell Cardiol.

[204] Ngoh GA, Papanicolaou KN, Walsh K (2012). Loss of mitofusin 2 promotes endoplasmic reticulum stress. J Biol Chem..

[205] Hall AR, Burke N, Dongworth RK (2016). Hearts deficient in both Mfn1 and Mfn2 are protected against acute myocardial infarction. Cell Death Dis..

[206] Piquereau J, Caffin F, Novotova M (2012). Down-regulation of OPA1 alters mouse mitochondrial morphology, PTP function, and cardiac adaptation to pressure overload. Cardiovasc Res..

[207] Jiang X, Jiang H, Shen Z, Wang X (2014). Activation of mitochondrial protease OMA1 by Bax and Bak promotes cytochrome c release during apoptosis. Proc Natl Acad Sci U S A..

[208] Korwitz A, Merkwirth C, Richter-Dennerlein R (2016). Loss of OMA1 delays neurodegeneration by preventing stress-induced OPA1 processing in mitochondria. J Cell Biol..

[209] Xiao X, Hu Y, Quirós PM, Wei Q, López-Otín C, Dong Z (2014). OMA1 mediates OPA1 proteolysis and mitochondrial fragmentation in experimental models of ischemic kidney injury. Am J Physiol Renal Physiol..

[210] Ong S-B, Hall AR, Dongworth RK (2015). Akt protects the heart against ischaemia-reperfusion injury by modulating mitochondrial morphology. Thromb Haemost..

[211] Pyakurel A, Savoia C, Hess D, Scorrano L (2015). Extracellular regulated kinase phosphorylates mitofusin 1 to control mitochondrial morphology and apoptosis. Mol Cell..

[212] Sooyeon L, Go KL, Kim J-S (2015). Deacetylation of mitofusin-2 by sirtuin-1: A critical event in cell survival after ischemia. Mol Cell Oncol..

[213] Biel TG, Lee S, Flores-Toro JA (2016). Sirtuin 1 suppresses mitochondrial dysfunction of ischemic mouse livers in a mitofusin 2-dependent manner. Cell Death Differ..

[214] Cellier L, Tamareille S, Kalakech H (2016). Remote ischemic conditioning influences mitochondrial dynamics. Shock..

[215] Varanita T, Soriano ME, Romanello V (2015). The opa1-dependent mitochondrial cristae remodeling pathway controls atrophic, apoptotic, and ischemic tissue damage. Cell Metab..

[216] Chang Y-W, Chang Y-T, Wang Q, Lin JJ-C, Chen Y-J, Chen C-C (2013). Quantitative phosphoproteomic study of pressure-overloaded mouse heart reveals dynamin-related protein 1 as a modulator of cardiac hypertrophy. Mol Cell Proteomics..

[217] Fang L, Moore X-L, Gao X-M, Dart AM, Lim YL, Du X-J (2007). Down-regulation of mitofusin-2 expression in cardiac hypertrophy in vitro and in vivo. Life Sci..

[218] Yu H, Guo Y, Mi L, Wang X, Li L, Gao W (2011). Mitofusin 2 inhibits angiotensin II-induced myocardial hypertrophy. J Cardiovasc Pharmacol Ther..

[219] Marechal X, Montaigne D, Marciniak C (2011). Doxorubicin-induced cardiac dysfunction is attenuated by ciclosporin treatment in mice through improvements in mitochondrial bioenergetics. Clin Sci (Lond)..

[220] Chalmers S, Saunter C, Wilson C, Coats P, Girkin JM, McCarron JG (2012). Mitochondrial motility and vascular smooth muscle proliferation. Arterioscler Thromb Vasc Biol..

[221] Lim S, Lee S-Y, Seo H-H (2015). Regulation of mitochondrial morphology by positive feedback interaction between PKCδ and Drp1 in vascular smooth muscle cell. J Cell Biochem..

[222] Maimaitijiang A, Zhuang X, Jiang X, Li Y (2016). Dynamin-related protein inhibitor downregulates reactive oxygen species levels to indirectly suppress high glucose-induced hyperproliferation of vascular smooth muscle cells. Biochem Biophys Res Commun..

[223] Zhang L, Ma C, Zhang C (2016). Reactive oxygen species effect PASMCs apoptosis via regulation of dynamin-related protein 1 in hypoxic pulmonary hypertension. Histochem Cell Biol..

[224] Chen K-H, Guo X, Ma D (2004). Dysregulation of HSG triggers vascular proliferative disorders. Nat Cell Biol..

[225] Zhou W, Chen K-H, Cao W (2010). Mutation of the protein kinase A phosphorylation site influences the anti-proliferative activity of mitofusin 2. Atherosclerosis..

[226] Ding Y, Gao H, Zhao L, Wang X, Zheng M. Mitofusin 2-deficiency suppresses cell proliferation through disturbance of autophagy. Zhang J, ed. PLoS One. 2015;10:e0121328. 10.1371/journal.pone.0121328 PMC4363693 25781899

[227] Fang X, Chen X, Zhong G, Chen Q, Hu C (2016). Mitofusin 2 downregulation triggers pulmonary artery smooth muscle cell proliferation and apoptosis imbalance in rats with hypoxic pulmonary hypertension via the PI3K/Akt and mitochondrial apoptosis pathways. J Cardiovasc Pharmacol..

[228] Sharp WW, Beiser DG, Fang YH (2015). Inhibition of the mitochondrial fission protein dynamin-related protein 1 improves survival in a murine cardiac arrest model. Crit Care Med..

[229] Sumida M, Doi K, Ogasawara E (2015). Regulation of mitochondrial dynamics by dynamin-related protein-1 in acute cardiorenal syndrome. J Am Soc Nephrol..

[230] Wang Y, Katayama A, Terami T (2015). Translocase of inner mitochondrial membrane 44 alters the mitochondrial fusion and fission dynamics and protects from type 2 diabetes. Metabolism..

[231] Montaigne D, Marechal X, Coisne A (2014). Myocardial contractile dysfunction is associated with impaired mitochondrial function and dynamics in type 2 diabetic but not in obese patients. Circulation..

[232] Liu R, Jin P, Yu L (2014). Impaired mitochondrial dynamics and bioenergetics in diabetic skeletal muscle. PLoS One..

[233] Reinhardt F, Schultz J, Waterstradt R, Baltrusch S (2016). Drp1 guarding of the mitochondrial network is important for glucose-stimulated insulin secretion in pancreatic beta cells. Biochem Biophys Res Commun..

[234] Yu P, Zhang J, Yu S (2015). Protective effect of sevoflurane postconditioning against cardiac ischemia/reperfusion injury via ameliorating mitochondrial impairment, oxidative stress and rescuing autophagic clearance. PLoS One..

[235] Bucha S, Mukhopadhyay D, Bhattacharyya NP (2015). Regulation of mitochondrial morphology and cell cycle by microRNA-214 targeting Mitofusin2. Biochem Biophys Res Commun..

[236] Hull TD, Boddu R, Guo L (2016). Heme oxygenase-1 regulates mitochondrial quality control in the heart. JC Iinsight..

[237] Veeranki S, Givvimani S, Kundu S, Metreveli N, Pushpakumar S, Tyagi SC (2016). Moderate intensity exercise prevents diabetic cardiomyopathy associated contractile dysfunction through restoration of mitochondrial function and connexin 43 levels in db/db mice. J Mol Cell Cardiol..

[238] Zhang Q, Wu Y, Zhang P, Sha H, Jia J, Hu YZJ (2012). Exercise induces mitochondrial biogenesis after brain ischemia in rats. Neurosci..

[239] Suwanjang W, Abramov AY, Charngkaew K, Govitrapong P, Chetsawang B (2016). Melatonin prevents cytosolic calcium overload, mitochondrial damage and cell death due to toxically high doses of dexamethasone-induced oxidative stress in human neuroblastoma SH-SY5Y cells. Neurochem Int..

[240] Pei H, Du J, Song X (2016). Melatonin prevents adverse myocardial infarction remodeling via Notch1/Mfn2 pathway. Free Radic Biol Med..

[241] Reshma PL, Sainu NS, Mathew AK, Raghu KG (2016). Mitochondrial dysfunction in H9c2 cells during ischemia and amelioration with Tribulus terrestris L. Life Sci..

[242] Yin X, Manczak M, Reddy PH (2016). Mitochondria-targeted molecules MitoQ and SS31 reduce mutant huntingtin-induced mitochondrial toxicity and synaptic damage in Huntington’s disease. Hum Mol Genet..

[243] Mourier A, Motori E, Brandt T (2015). Mitofusin 2 is required to maintain mitochondrial coenzyme Q levels. J Cell Biol..

[244] Li A, Zhang S, Li J, Liu K, Huang F, Liu B (2016). Metformin and resveratrol inhibit Drp1-mediated mitochondrial fission and prevent ER stress-associated NLRP3 inflammasome activation in the adipose tissue of diabetic mice. Mol Cell Endocrinol..

[245] Torres G, Morales PE, García-Miguel M (2016). Glucagon-like peptide-1 inhibits vascular smooth muscle cell dedifferentiation through mitochondrial dynamics regulation. Biochem Pharmacol..

[246] Chen H, Ren S, Clish C (2015). Titration of mitochondrial fusion rescues Mff-deficient cardiomyopathy. J Cell Biol..

